# Gut microbiome resistotypes reflect chronological age and *Enterobacteriaceae* relative abundance

**DOI:** 10.3389/fmicb.2026.1869983

**Published:** 2026-09-30

**Authors:** Elizaveta V. Starikova, Polina A. Kuzmichenko, Dmitry E. Fedorov, Andrey V. Vvedensky, Alexander V. Pavlenko, Anna L. Arkhipova, Svetlana N. Kovalchuk, Elena N. Ilina

**Affiliations:** 1 Martsinovsky Institute of Medical Parasitology, Tropical and Vector Borne Diseases, Sechenov University, Moscow, Russia; 2 Research Institute of Systems Biology and Medicine (RISBM), Moscow, Russia

**Keywords:** aging, antibiotic resistance, COVID-19, resistome, resistotypes

## Abstract

**Background:**

Surveillance of antibiotic resistance genes (ARGs) in the human gut microbiota is critical for informing public health interventions and antibiotic stewardship strategies, taking into account sociodemographic, behavioral, and health-related factors. Although metagenomics research has grown exponentially, few studies have described resistome dynamics over time. In this study, we investigated resistome profiles over a 5-year interval (between 2016-2017 and 2022) in adult residents of Arkhangelsk, Northwestern Russia.

**Results:**

Over 5 years, we observed an increase in ARGs richness and abundance along with the prevalence changes in genes conferring resistance to aminoglycosides (*aph2, aph3, ant9*), lincosamides (*lnuC*), chloramphenicol (*cat, catA*), and macrolides (*ermG, ermB*). We have identified two resistotypes in our data that differed in the number of identified ARGs. The less frequent resistotype 2, characterized by a higher number of ARGs, was associated with higher participants' chronological age and increased *Enterobacteriaceae* abundance. Approximately one-third of the participants have shifted from one resistotype to another within a 5-year period. Temporal changes in resistome profiles occurred unevenly among participants, with the greatest changes noted for 41-45 and 61-65 age groups. No associations were found between COVID-19 history and resistome composition.

**Conclusions:**

We showed an increase in overall ARG richness, ARG abundance, and the prevalence of certain ARGs in microbiomes of adult residents of Arkhangelsk over a 5-year period. We have identified two resistotypes; the one with higher ARGs richness was linked to higher chronological age and *Enterobacteriaceae* family relative abundance. These findings underscore the necessity for further antibiotic resistance surveillance to address the accelerating global dissemination of ARGs, while highlighting age as an underrecognized factor contributing to resistome dynamics.

## Introduction

Antibiotic resistance is a major public health challenge. Infections caused by multidrug resistant bacteria often fail to respond to treatment, resulting in prolonged illness, increased risk of complications and death, and higher healthcare costs (World Health Organization, 2023). An estimated 4.71 million deaths were associated with antibiotic resistance in 2021, including 1.14 million deaths attributed to antibiotic resistance directly (Naghavi et al., 2024). Global predictions indicate that by 2050, the number of deaths directly attributable to antibitic resistance could reach 1.91 millions, with 8.22 million deaths associated with it (Naghavi et al., 2024).

The spread of antibiotic resistance is driven by antibiotic resistance genes (ARGs) that confer resistance through several mechanisms, such as antibiotic inactivation, drug target modifications, altering cell wall permeability, and efflux pumps. The human gut microbiota acts as a key reservoir for ARGs, where these genes accumulate and are exchanged between bacteria. Many human gut commensals can incorporate foreign DNA, including ARGs (Yan et al., 2022), which can be subsequently transferred to other bacterial species, including pathogenic ones (Karami et al., 2007).

The collection of these genes within microbial community, known as the resistome (Van Schaik, 2015), is influenced by antibiotic exposure and host-related factors including age (Wu et al., 2021; Tavella et al., 2021), diet (Oliver et al., 2022), chronic diseases (Pan et al., 2022), healthcare use (Pan et al., 2022), international travel (Worby et al., 2023), and environmental exposures (Van Gompel et al., 2020; Li et al., 2021). Resistome profiles can be country-specific due to differing antibiotic policies and healthcare practices (Xia et al., 2019). Metagenomic studies have shown that resistome profiles across individuals often form distinct clusters, referred to as resistotypes, which differ in ARG content and abundance patterns (Pavlenko et al., 2025; Lee et al., 2023; Starikova et al., 2024).

The global burden of antimicrobial resistance intensified during the COVID-19 pandemic due to the widespread empirical antibiotic use (Rizvi and Ahammad, 2022). Studies of COVID-19-recovered individuals with antibiotic history show increased gut ARGs relative abundance and diversity, alongside higher prevalence of mobile genetic elements (Kang et al., 2022).

Monitoring the gut resistome is essential to inform public health strategies against antimicrobial resistance and to support antibiotic stewardship, particularly in the context of gut dysbiosis and its association with sociodemographic, behavioral, and health-related factors. A particular concern is the ubiquitous presence of “core” ARGs (e.g. *tetM, ermB*) found in nearly all individuals (Starikova et al., 2024; Feng et al., 2018). This highlights the need for longitudinal analyses of resistomes within populations to monitor the spread of ARGs. While human gut resistomes are well-characterized, existing longitudinal studies are scarce and focus primarily on short-term effects following antibiotic exposure (Willmann et al., 2019). A long-term longitudinal design enables detection of population-level changes in resistome composition, providing data for identifying specific resistance determinants that may pose future public health threats. To date, however, resistome changes in healthy populations over extended periods remain unexplored.

To address this gap, we investigated the resistome profiles of the adult residents of Arkhangelsk, Northwestern Russia over a 5-year period from 2016-2017 to 2022, which included the period of the COVID-19 pandemic.

## Materials and methods

###  Study participants and metadata collection

A cohort study was conducted with adult residents of Arkhangelsk, Northwestern Russia, who provided paired stool samples collected 5 years apart. The study design is shown in Figure 1. The first sample set was collected in 2016-2017 as part of the population-based Know Your Heart (KYH) study Cook et al. (2018), while the second set was collected in the autumn of 2022 during a follow-up study assessing COVID-19 pandemic effects. Participants were followed up for COVID-19 diagnosis using the Federal Registry of COVID-19 Patients. Of the 333 participants providing follow-up samples in 2022, 175 with complete COVID-19 history data were selected. Overall, 350 samples from two study periods (2016-2017 and 2022) were analyzed.

**Figure 1:**
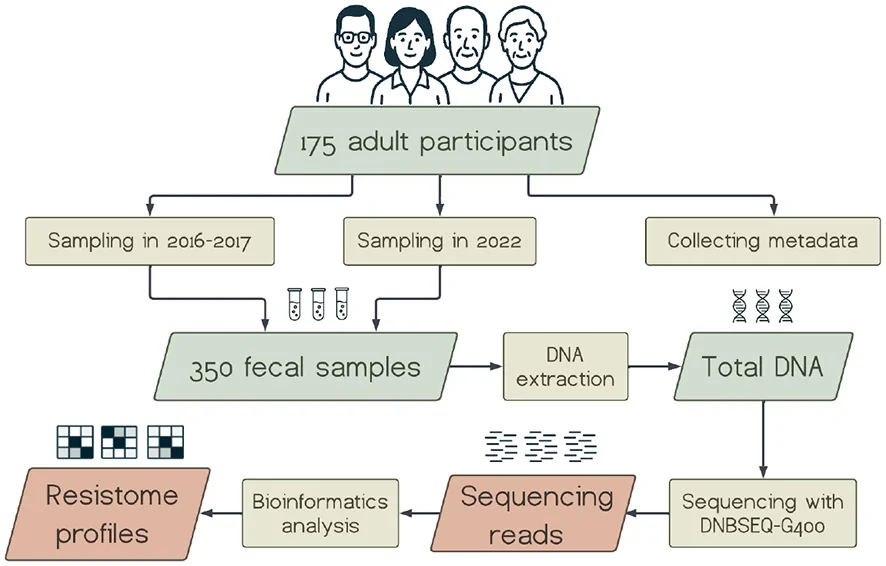
Study design

The KYH protocol included a physical examination and a questionnaires on sociodemographics (age, sex), lifestyle (diet quality, drinking frequency), and chronic diseases (arterial hypertension, ischemic heart disease, arrhythmia, rheumatoid arthritis, diabetes mellitus, asthma, chronic bronchitis, neoplasms, liver disease, kidney disease). Other details of KYH design are published elsewhere (Cook et al., 2018).

Participants were grouped by age (35-44, 45-59, and 60+ years) based on recent findings of aging trajectory shifts around 44 and 60 years Shen et al. (2024). Alcohol consumption was categorized into three levels: non-drinkers, moderate ( ≤ 1/week), or frequent (≥ 2/week) drinkers based on self-reported frequency. Dietary quality was assessed using the Dietary Quality Score (DQS) Score (Toft et al., 2007) and grouped as: unhealthy (DQS 1-3), average (DQS 4-6), or healthy diet (DQS 7-9). BMI was calculated as weight (kg) divided by the square of height (m^2^). The participants were categorized into three BMI groups: normal weight (BMI ≤ 25 kg/m^2^), overweight (25 < BMI ≤ 30 kg/m^2^), and obesity (BMI > 30 kg/m^2^). Participants were classified as having a chronic condition (yes/no) if they had at least one of the following: arterial hypertension, ischemic heart disease, arrhythmia, rheumatoid arthritis, diabetes mellitus, asthma, chronic bronchitis, neoplasm, liver disease, kidney disease, or obesity (BMI > 30kg/m^2^).

For the 2022 data, both COVID-19 diagnosis (yes/no) and hospitalization status (yes/no) were considered, as recorded in the Federal Registry of COVID-19 Patients.

A summary of the participants' metadata is provided in Supplementary Table 1.

###  Sample collection

We analyzed paired stool samples from 175 participants, self-collected at home across two timepoints: 2016-2017 and 2022 (350 samples in total). Participants avoided medications that could affect the gastrointestinal tract (enterosorbents, laxatives, enzymes, etc.) prior to stool collection. Samples were stored at 4°C for no more than 24 after defecation, then transported to the laboratory, and frozen at -80°C. Frozen samples were transported on dry ice at -50°C to a sequencing facility, where DNA was isolated and stored at -20°C until further analysis.

###  DNA extraction and sequencing

Stool DNA was isolated using the MagPure Stool DNA LQ Kit (Magen) per manufacturer's instructions, quantified via Qubit 3.0 fluorometer (Thermo Fisher Scientific) using Qubit dsDNA BR Assay, and 100 ng was used for library preparation (FastFS DNA Library Prep Set, MGI). DNA nanoballs were prepared using the DNBSEQ-G400RS kit (FCL PE150, MGI). High-throughput sequencing was conducted on the DNBSEQ-G400 (MGI) platform (2 x 150 bp 109 paired-end) in six batches. 2016-2017 and 2022 samples were distributed equally within each batch (Supplementary Figure 2). Paired samples were sequenced within the same batch, and eight technical controls were added to each run (Supplementary Table 2).

###  Metagenomic data processing and analysis

The bioinformatics analysis workflow is shown in Figure 2.

**Figure 2:**
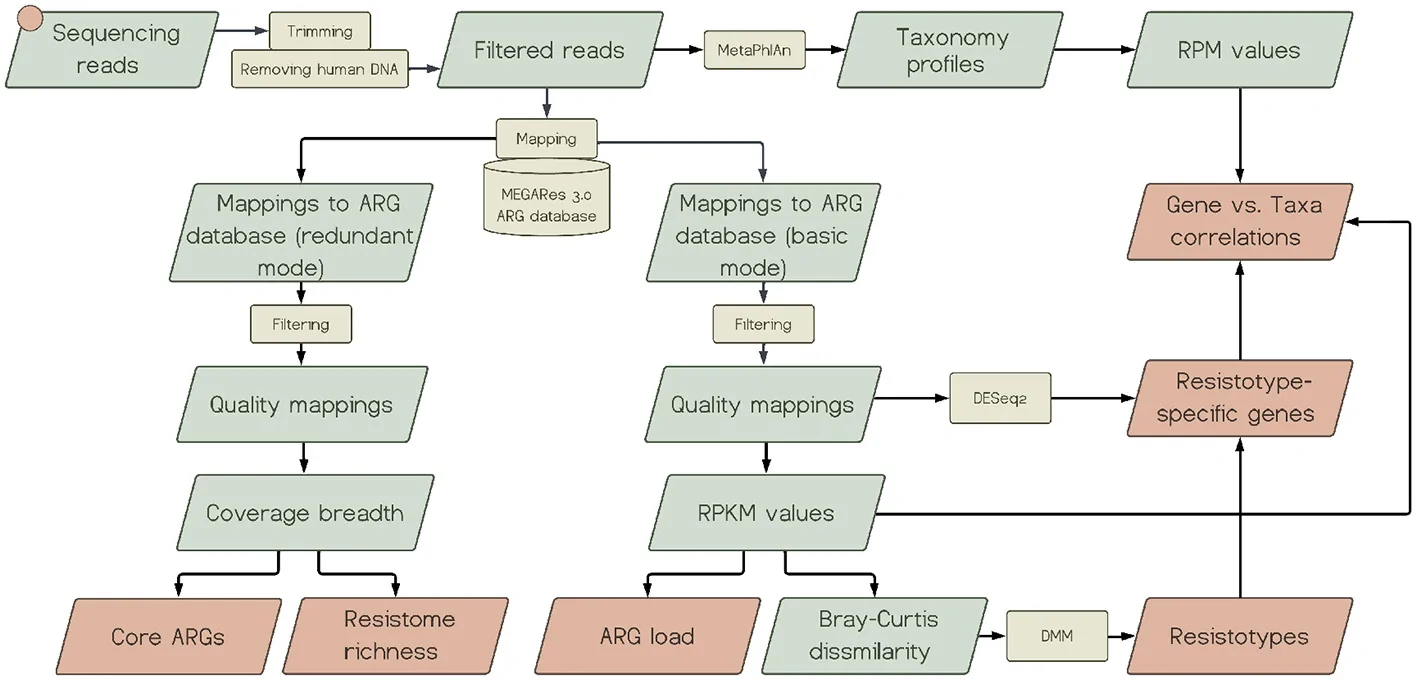
Bioinformatics workflow for metagenomic data processing and analysis

The resistome profiling pipeline is available at GitHub (https://github.com/bobeobibo/ResistoViper).

###  Quality control and decontamination

Adapters were excised with cutadapt (Martin, 2011), and quality filtering was performed using Trimmomatic (v.0.39-2) (Bolger et al., 2014) LEADING:25 TRAILING:25 SLIDINGWINDOW:4:15 MINLEN:50). Human DNA was filtered using biobloom https://github.com/bcgsc/biobloom/tree/master.

###  Resistome profiles

Resistome profiles were generated by mapping decontaminated reads to a subset of antibiotic resistance determinants (7,535 sequences, 967 genes; metal resistance genes excluded) from MEGARes 3.0 (https://www.meglab.org/megares/) using bowtie2 instrument (v.2.4.2) in two modes:

- Basic mode. Single best alignment per read (“–non-deterministic”). Used to calculate relative abundance values for each ARG as RPKM (Reads Per Kilobase Per Million Mapped Reads) (Starikova et al., 2024)
- Redundant mode. All valid alignments (“-a” option). Used to calculate coverage width

All the mappings required MAPQ ≥ 30. Coverage width per gene variant was calculated via bedtools v2.31.1. Results were then aggregated to the gene level: a gene was considered “fully covered” if ≥ 98% breadth coverage was achieved in ≥ 1 of its variants. ARGs richness was defined as the number of “fully covered” genes per sample. Prevalence of an ARG was defined as the proportion of samples where the gene was “fully covered”. RPKM values were calculated according to the following formula (1), summed across variants within each gene (Starikova et al., 2024):


$$\begin{array}{l} RPKM_{gene}=\frac{reads\;mapped\;to\;all\;sequence\;variants\;}{total\;reads\;in\;sample\;\left(mln\right)\cdot median\;sequence\;variant\;length\;of\;a\;gene\;\left(kbp\right)} \end{array}$$ (1)

ARG load was defined as the sum of all RPKM values per sample.

Temporal changes in these metrics were evaluated using the Wilcoxon signed-rank paired test. “Core” ARGs were defined as fully covered genes present in >50% of samples per timepoint. Prevalence changes were assessed with McNemar's test, *p*-values were adjusted using Bonferroni correction.

ARG richness and ARG load values were compared between groups using linear mixed-effect models (LMM) for variables with repeated measurements (age group, sex, BMI category) using the lmerTest R package (version 3.2-1). Each model included the respective variable and year as fixed effects, and participant ID as a random effect to account for repeated measurements. Pairwise comparisons were performed using estimated marginal means with Bonferroni correction using the emmeans R package (version 2.0.4). For variables measured at a single time point (diet quality, COVID-19 hospitalization, alcohol intake, chronic condition), Mann–Whitney *U*-test (two categories) or Kruskal-Wallis test (three categories) were applied. For multi-category variables, Mann–Whitney *U*-test was additionally performed to assess differences between selected group pairs.

For community-level analysis, Bray-Curtis dissimilarity indices were calculated between each pair of samples based on their RPKM values using vegan R package (v2.6-4). Within-group heterogeneity was assessed via PERMDISP to control for pseudo-replication. Potential clusters (“resistotypes”) were identified using Dirichlet-multinomial mixture models (DirichletMultinomial R package v1.40.0) based on RPKM values of all samples. The optimal number of clusters was determined by minimized Laplace, BIC (Bayesian Information Criterion) and AIC (Akaike Information Criterion) metrics. Differential ARG representation between resistotypes was analyzed using DESeq2 (v1.38.3) with a negative binomial model (design = ~ RT). Normalization was performed using the median-of-ratios method. Results were extracted with the Wald test and adjusted for multiple testing using Bonferroni correction (pAdjustMethod = “bonferroni”). Genes with adjusted *p*-value < 0.05 were considered differentially represented. Additionally, we used MaAsLin3 (v3.1.2) with total–sum scaling (normalization = “TSS”), log transformation (transform = “LOG”), and Bonferroni correction (correction = “bonferroni”). The model included resistotype as a fixed effect (formula = ~ RT). Significance was defined as *q*-value < 0.05 in the abundance model. ARGs richness and ARGs load values were compared between samples belonging to different resistotypes using Mann–Whitney *U*-test.

###  Taxonomy profiles

Taxonomic classification used MetaPhlAn v4.0.6 with the mpa_vOct22_CHOCOPhlAnSGB_202212 database (default parameters). Read counts per taxon were converted to relative abundance (RPM, Reads Per Million Mapped Reads) for cross-sample comparability (2):


$$\begin{array}{l} RPM_{taxon}=10^{6}\cdot \frac{reads\;assigned\;to\;this\;taxon}{total\;reads\;in\;a\;sample} \end{array}$$ (2)

###  Connecting resistome data with taxonomy data

Spearman correlations between 65 RT2-specific ARGs (RPKM) and bacterial taxa (RPM) were calculated using Hmisc (v5.1-3). Rare taxa and rare genes (< 10% prevalence) were excluded. Gene-gene and gene-taxon pairs with ρ> 0.7 and adjusted *p*-value < 0.050 were selected for family, genus and species levels. RPM values of the selected taxa were compared between resistotypes (Mann–Whitney *U*-test).

###  Connecting resistome and taxonomy data with the participants' metadata

ARG richness, ARG load, Bray-Curtis dissimilarity, and selected taxa RPM values were compared between groups using linear mixed-effects models (LMM) for variables measured at both time points (age group, sex, BMI category). The models included the respective variable and year as fixed effects, and participant ID as a random effect. Pairwise comparisons were performed using estimated marginal means with Bonferroni correction. For variables measured at a single time point (chronic condition, diet quality, alcohol intake, COVID-19 hospitalization), Mann–Whitney *U*-test or Kruskal–Wallis test were applied. Bray-Curtis dissimilarity was also compared across 5-year age groups. For multi-category variables, Mann–Whitney *U*-test was additionally performed to assess differences between selected group pairs.

Resistotype proportions were compared within selected categories using Chi-squared test.

## Results

###  Metagenomic sequencing

Metagenomic samples were sequenced on the DNBSEQ-G400 (MGI) platform. Data was generated for 350 samples, with a median of 46.2 mln paired reads per sample (range: 6.8-115.2 mln reads, Supplementary Table 3). Rarefaction analysis confirmed that sequencing depth was sufficient for ARG richness estimation (Supplementary Figure 1). PERMANOVA analysis (Bray-Curtis, 999 permutations) on genus-level taxonomic composition confirmed no significant batch effect between runs (*R*^2^ = 0.009, *p* = 0.896, Supplementary Figure 3). We found no significant confounding effects of sequencing run, DNA concentration, or sequencing depth on ARG richness and load (Supplementary Figure 4).

###  Resistome composition and dynamics over time

#### Prevalence of multiple ARGs increased significantly

A total of 222 unique ARGs were detected across all 350 samples. We first analyzed the prevalence of these genes across the cohort. ARGs detected in >50% of samples per sample set were defined as “core”. Using McNemar's test for each of the genes in the subset, we have identified eight genes that significantly differed in prevalence between 2016-2017 and 2022: aminoglycoside O-nucleotidyltransferase (*ant9*); aminoglycoside O-phosphotransferases (*aph3, aph2*); lincosamide nucleotidyltransferase (*lnuC*); chloramphenicol acetyltransferases (*cat, catA*); and 23S rRNA methyltransferases (*ermG, ermB*) conferring resistance to macrolides (Figure 3, Supplementary Table 4). All the eight genes became more prevalent over 5 years, with certain genes reaching up to 99% of samples in 2022 (*ermB* gene). *TetO* and *tetW* tetracycline resistance genes remained at 100% prevalence throughout.

**Figure 3:**
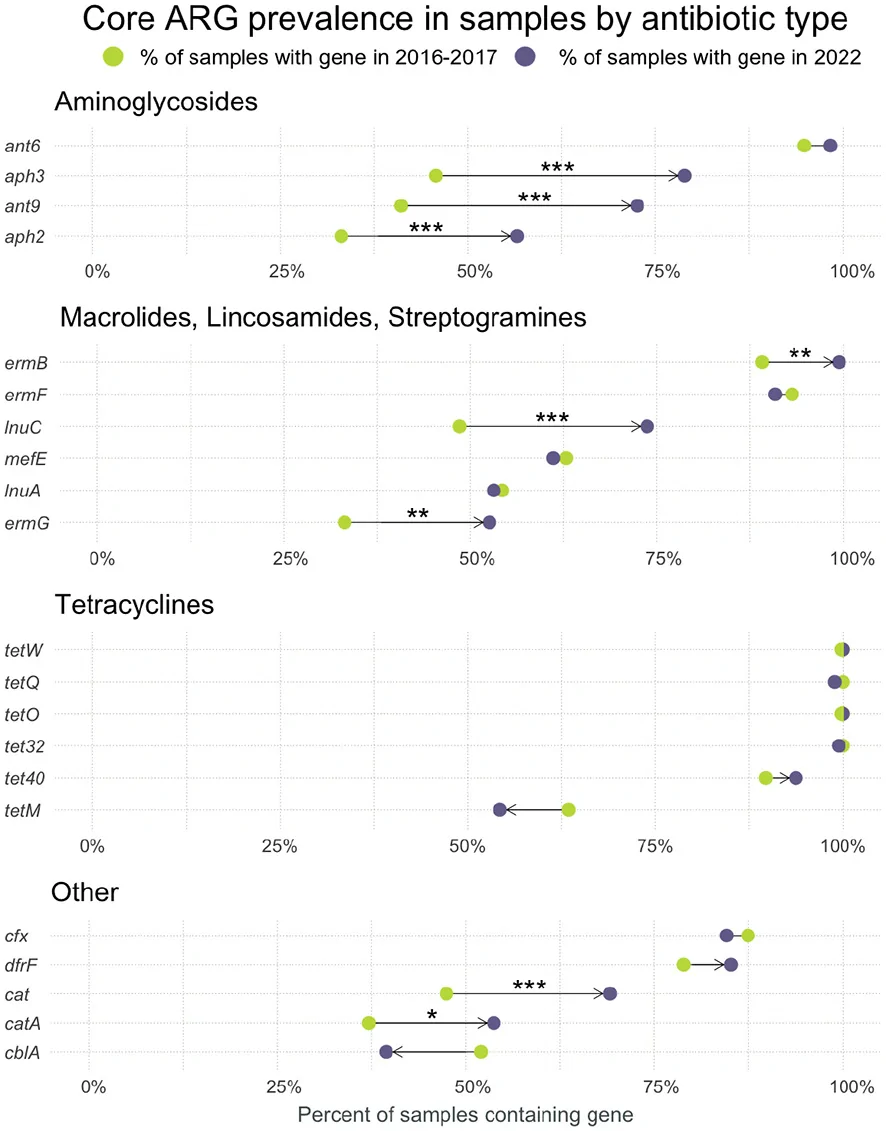
Dumbbell chart illustrating the changes in core ARGs prevalence over time. Genes that showed a significant change in prevalence between years, according to McNemar's test, are marked with asterisks: ***p-value < 0.001, **p-value < 0.010, *p-value < 0.05. Arrows indicate the direction of prevalence change. The set of genes is a union of 2016-2017 ARGs core and 2022 ARGs core.

Analysis of non-core ARGs identified significant prevalence changes for 69 of 199 genes (Supplementary Table 5), most of which were efflux pump genes associated with *Enterobacteriaceae*; others included aminoglycoside acetyltransferase *aac6*, streptothricin acetyltransferase *sat* and tetracycline resistance gene *tetBP*. All except *tetBP* increased in prevalence in 2022.

Overall, 77 genes have changed prevalence over 5 years.

#### ARGs richness and load increased significantly

We next quantified the overall burden of antibiotic resistance. The median ARGs richness value across all samples was 23 (Supplementary Figure 5, also see Supplementary Table 6 for a summary of ARGs detected in all samples). A significant increase was observed between time periods (Wilcoxon signed-rank test *p*-value < 0.001), with the median number of ARGs rising from 21 to 27.

The median ARGs load value was 167 (Supplementary Figure 6), also increasing significantly between time periods (Wilcoxon signed-rank test *p*-value < 0.001).

This indicates a clear trend of rising ARGs diversity and relative abundance.

#### Resistome dissimilarity increased over time

To assess individual resistome changes, we calculated Bray-Curtis dissimilarity between sample pairs using ARGs RPKM values. The median Bray-Curtis index between paired samples of the same participant was 0.56 (range 0.14-0.98). While samples from the same individual remained more similar to each other than to samples from other individuals (Supplementary Figures 7a–c), the overall dissimilarity between samples from all participants differed significantly between 2016-2017 and 2022 sample sets (Wilcoxon signed-rank test p-value < 0.001, effect size = 0.04, Supplementary Figures 7d, e), indicating increasing resistome heterogeneity within population.

#### One-third of participants switched resistotypes within 5 years

To identify potential resistotypes, we have performed clustering using Dirichlet-multinomial mixture models based on RPKM values of all the samples. Standard model selection metrics (AIC, BIC, Laplace) supported two distinct resistotypes (Supplementary Figures 8a, 9). Overall, 261 samples were assigned to resistotype 1 (RT1), and 89 samples were assigned to resistotype 2 (RT2). Notably, the 2022 sample set had a higher proportion of RT2 samples.

RT2 showed significantly higher ARGs richness and load than RT1 (Mann–Whitney *U*-test *p*-values < 0.001, Figures 4, 5A).

**Figure 4:**
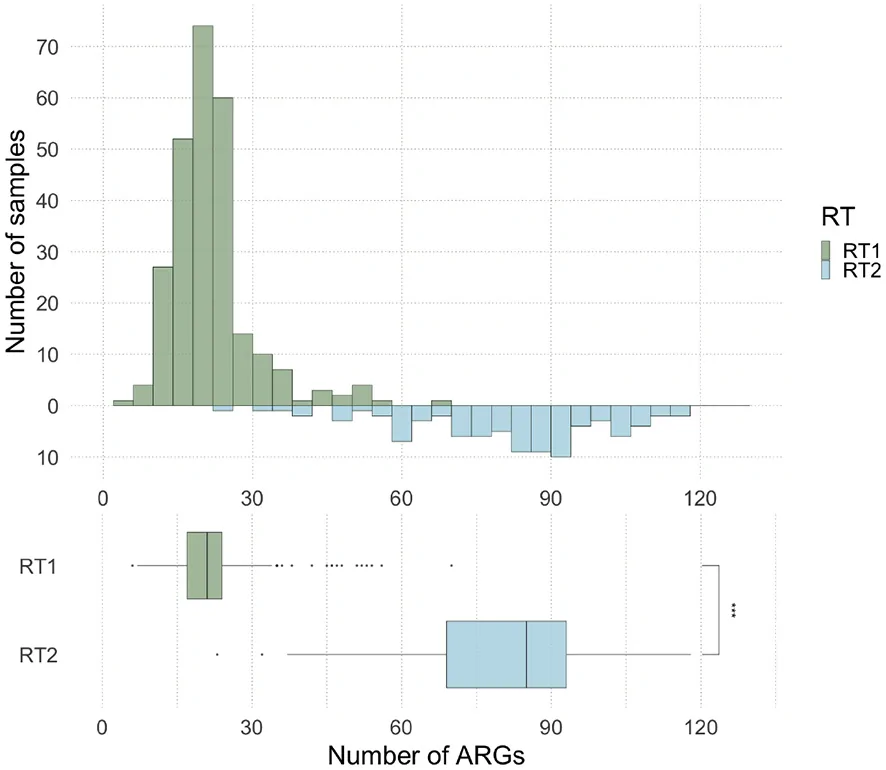
Distribution of the number of ARGs in each resistotype: RT1 – resistotype 1 (shown in green) and RT2 – resistotype 2 (shown in blue). ***Mann–Whitney *U*-test *p*-value < 0.001

**Figure 5:**
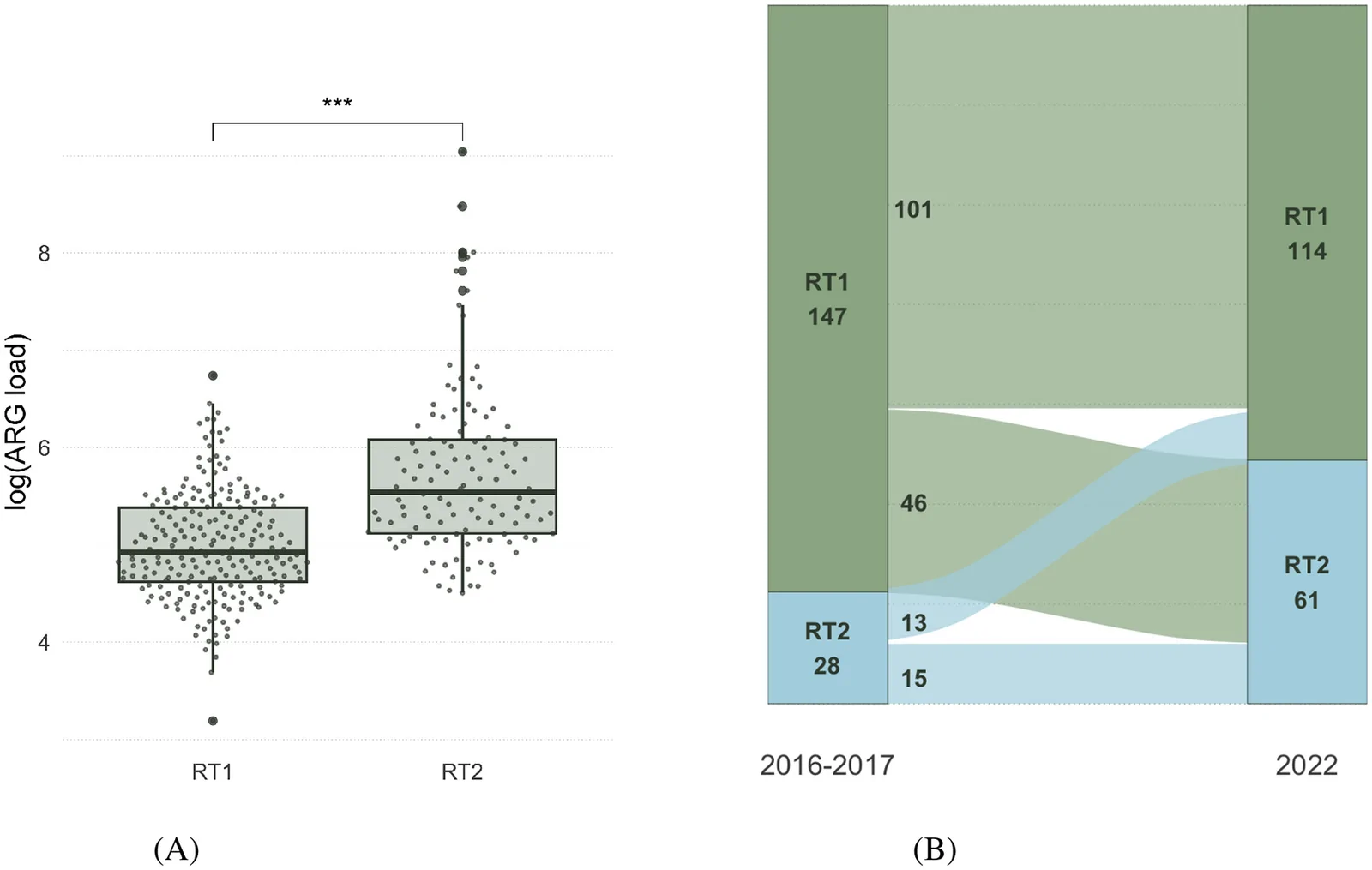
**(A)** Boxplots representing ARG load distributions (log-transformed) in samples belonging to RT1 (*n* = 261) and RT2 (*n* = 89). ***Mann–Whitney *U*-test *p*-value < 0.001. Dots represent individual samples. **(B)** Alluvial plot showing resistotype transitions from 2016-2017 to 2022.

When analyzing resistotype distributions in each sample set, we found that 59 (59/175, 34%) participants have changed their initial resistotype during a 5-year span. Of those, 46 (46/147, 31%) participants who had RT1 in 2016-2017 have transited to RT2 within 5 years, and 13 (13/28, 46%) participants who had RT2 in 2016-2017 have transited to RT1, correspondingly (Figure 5B).

DESeq2 identified 65 ARGs significantly overrepresented in RT2 (Supplementary Figure 10), including efflux pump genes, beta-lactamases (*ges, tem, blaEC, ctx, ampC*), and penicillin binding proteins (*apmH, pbp2, pbp4B*). No ARGs overrepresented in RT1 were identified. These findings were largely confirmed by MaAsLin3, which detected 54 RT2-specific ARGs, all of which overlapped with the DESeq2 set (Supplementary Figure 11). Additionally, MaAsLin3 identified one ARG (*tetQ*) as overrepresented in RT1, which was not detected by DESeq2.

###  Resistome features associate with age, BMI, and *Enterobacteriaceae* relative abundance

We assessed associations between resistome features (ARGs richness, ARGs load, Bray-Curtis dissimilarity, resistotype proportions) and the participants' metadata.

No associations were found between ARGs load/richness and sex, the presence of a chronic condition, diet quality, or alcohol intake (Supplementary Figures 12, 13). A significant association was found between BMI category and ARG load: participants in the Obese category had higher ARG load compared to the Overweight category (adjusted *p*-value = 0.04; LMM; Supplementary Figure 13b). A trend toward higher mean ARG richness was observed in participants with a history of COVID-19 hospitalization (45 genes vs. 36 genes), although this difference did not reach statistical significance (Mann–Whitney *U*-test *p*-value = 0.063; Supplementary Figure 12e). The smaller sample size of the hospitalized cohort (20 vs. 155 non-hospitalized) may have limited our ability to detect a significant association.

ARG richness and ARG load were both significantly associated with age. ARG richness was higher in participants aged 60+ compared to both younger age groups (adjusted *p*-values = 0.03 and 0.01 for 60+ vs. 35–44 and 60+ vs. 45–59, correspondingly; LMM; Figure 6A). For ARG load, a significant difference was observed only between the 60+ and 45–59 groups (adjusted *p*-value = 0.04; LMM; Figure 7B).

**Figure 6:**
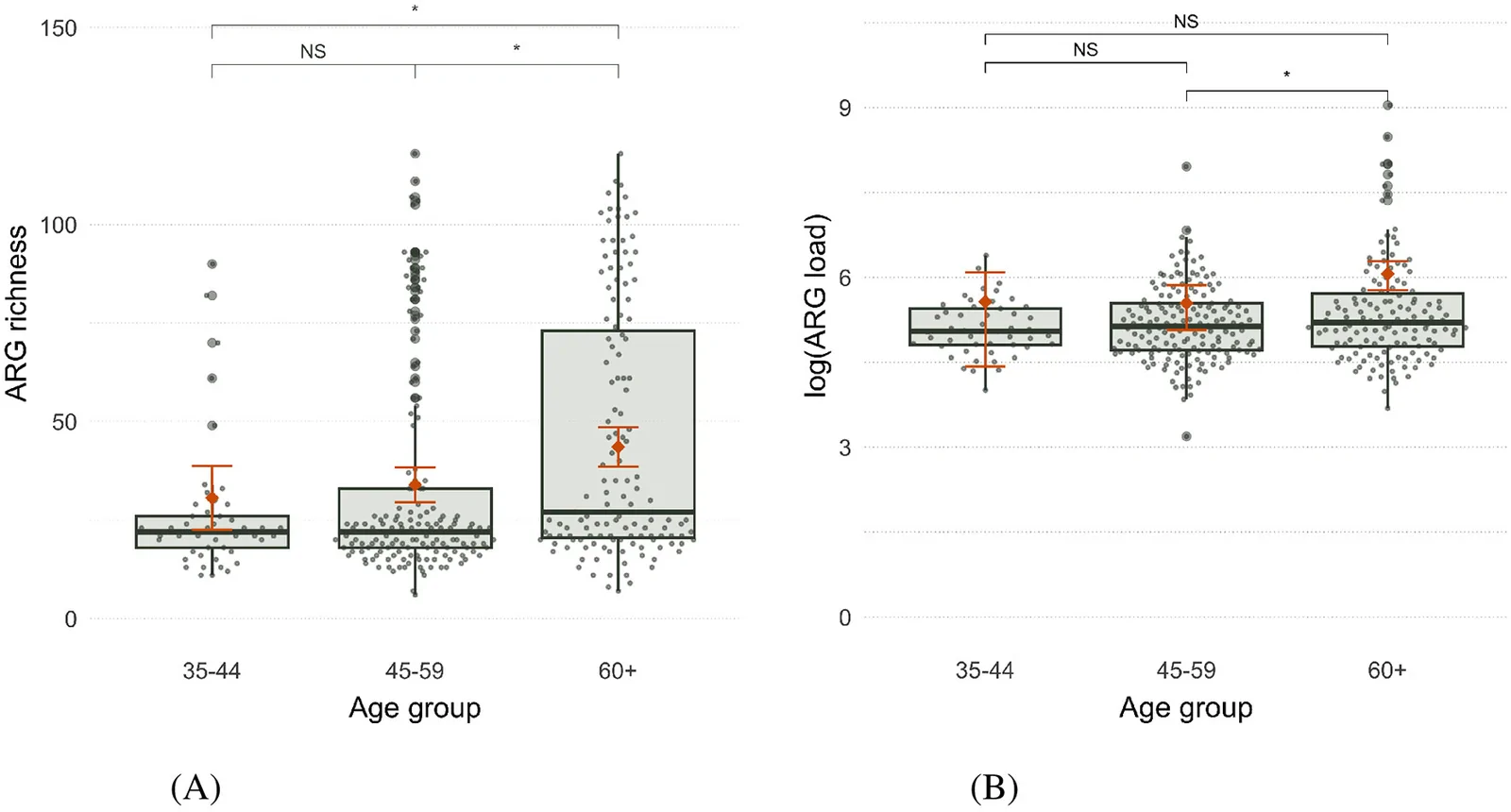
**(A)** ARG richness; **(B)** ARG load (log-transformed. ARGs richness and ARG load in relation to age groups. Orange points and error bars represent estimated marginal means with 95% confindence interval from the LMM (age group and year included as fixed effects; participant ID included as random effect). Significance levels (Bonferroni-corrected pairwise comparisons): *p < 0.05, NS – p > 0.05. Dots represent individual samples (*n* = 350).

**Figure 7:**
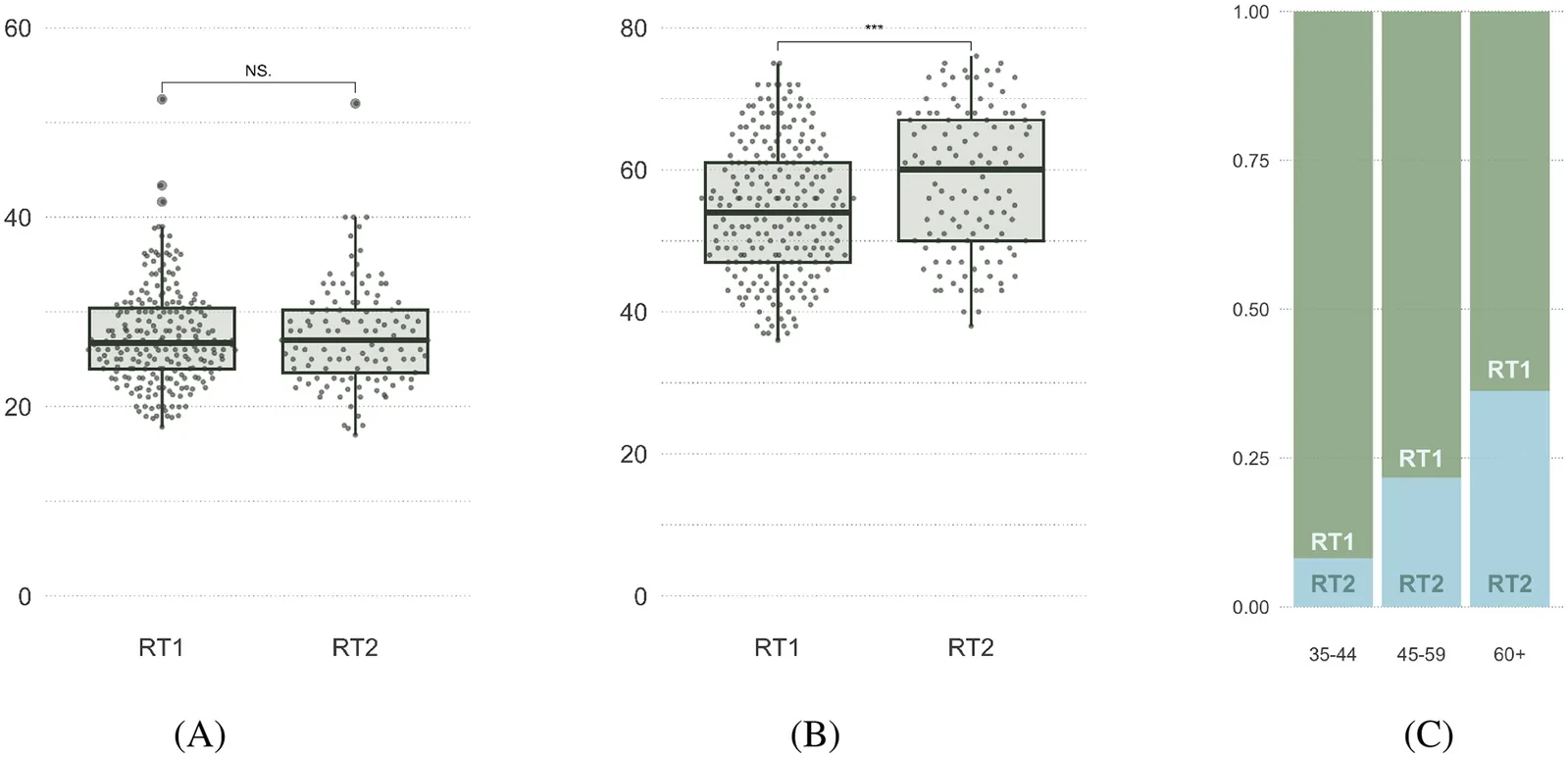
Resistotypes in relation to participants' metadata such as: **(A)** BMI, **(B)** chronological age, and **(C)** age group. Dots on boxplots represent individual samples. NS. – Mann–Whitney *U*-test not significant. ***Mann–Whitney *U*-test *p*-value < 0.001. Barplots represent proportions of all samples within age categories.

Analysis of Bray-Curtis dissimilarity between paired samples revealed a noticeable positive association with the participants' BMI (Supplementary Figure 14a). We also found that the participants reporting an unhealthy diet at baseline showed lower Bray-Curtis dissimilarity compared to those with healthy or average diets (Mann–Whitney *U*-test *p*-value = 0.054 and 0.037). No significant associations were found with sex, the presence of a chronic condition, alcohol intake, or broad baseline age groups (Supplementary Figures 14, 15). However, analysis using 5-year age intervals revealed heterogeneous distribution across groups, suggesting differential resistome variation with age (Supplementary Figure 15c). Notably, the 61-65 age group demonstrated the highest median Bray-Curtis dissimilarity index (0.67), differing from the 56-60, 51-55 and 46-50 age groups (Mann–Whitney *U*-test *p*-values = 0.030, 0.063 and 0.0049). The 41-45 age group had the second-highest median (0.63) differing significantly from the 46-50 age group (Mann–Whitney *U*-test *p*-value = 0.037).

These results indicate uneven resistome changes over 5 years across BMI and age groups. In this context, we further analyzed BMI and age in relation to the identified resistotypes. No significant BMI difference was found between participants with RT1 and RT2 (Mann–Whitney *U*-test *p*-value = 0.150, Figure 7A). However, participants with the assigned RT2 were significantly older than those with RT1 (median 60 vs. 55 years, Mann–Whitney *U*-test *p*-value < 0.001, Figure 7B). Resistotype proportions differed significantly between age groups (Chi-squared test *p*-value < 0.001), with the proportions of samples with assigned RT2 gradually increasing with age (Figure 7C). No associations were found between resistotypes and sex, BMI category, the presence of a chronic condition, diet quality, alcohol intake frequency, or COVID-19 hospitalization (Chi-squared test *p*-values > 0.050).

Participants transitioning from RT1 to RT2 over 5–6 years had a higher median age (55 years) than those maintaining RT1 (51 years), though not significantly (Mann–Whitney *U*-test *p*-value = 0.107, Supplementary Figure 16a). The age of the participants undergoing any type of resistotype transition (RT1-RT2 or RT2-RT1) did not differ significantly from those maintaining their initial resistotype (Mann–Whitney *U*-test *p*-value = 0.159, Supplementary Figure 16b). No associations were found between resistotype dynamics and other metadata.

To estimate the associations between resistotypes and bacterial taxa, we calculated Spearman's correlation coefficients for the 65 genes overrepresented in resistotype 2 and bacterial taxa on family, genus and species levels. This analysis identified a cluster of 53 co-abundant ARGs that highly correlated with the relative abundance of bacteria belonging to the *Enterobacteriaceae* family, and to *Escherichia coli* species in particular (Supplementary Figures 17–19, Supplementary Table 7).

*Enterobacteriaceae* relative abundance was significantly higher in RT2 samples compared to RT1 (median RPM values: 8,024 vs. 385, Mann–Whitney *U*-test *p*-value < 0.001, Figure 8A). Consistent with the resistotype-age association, *Enterobacteriaceae* relative abundance was significantly higher in participants of 60+ years of age compared to the 45-59 age group (adjusted *p*-value = 0.03; LMM; Figure 8B). No associations were found between sex, BMI category, diet quality, alcohol intake, the presence of a chronic condition, or COVID-19 hospitalization (Supplementary Figure 20). Furthermore, *Enterobacteriaceae* relative abundance differed significantly between 2016-2017 and 2022 time periods (Wilcoxon signed-rank paired test *p*-value < 0.001), with higher RPM values in 2022 (Figure 8C).

**Figure 8:**
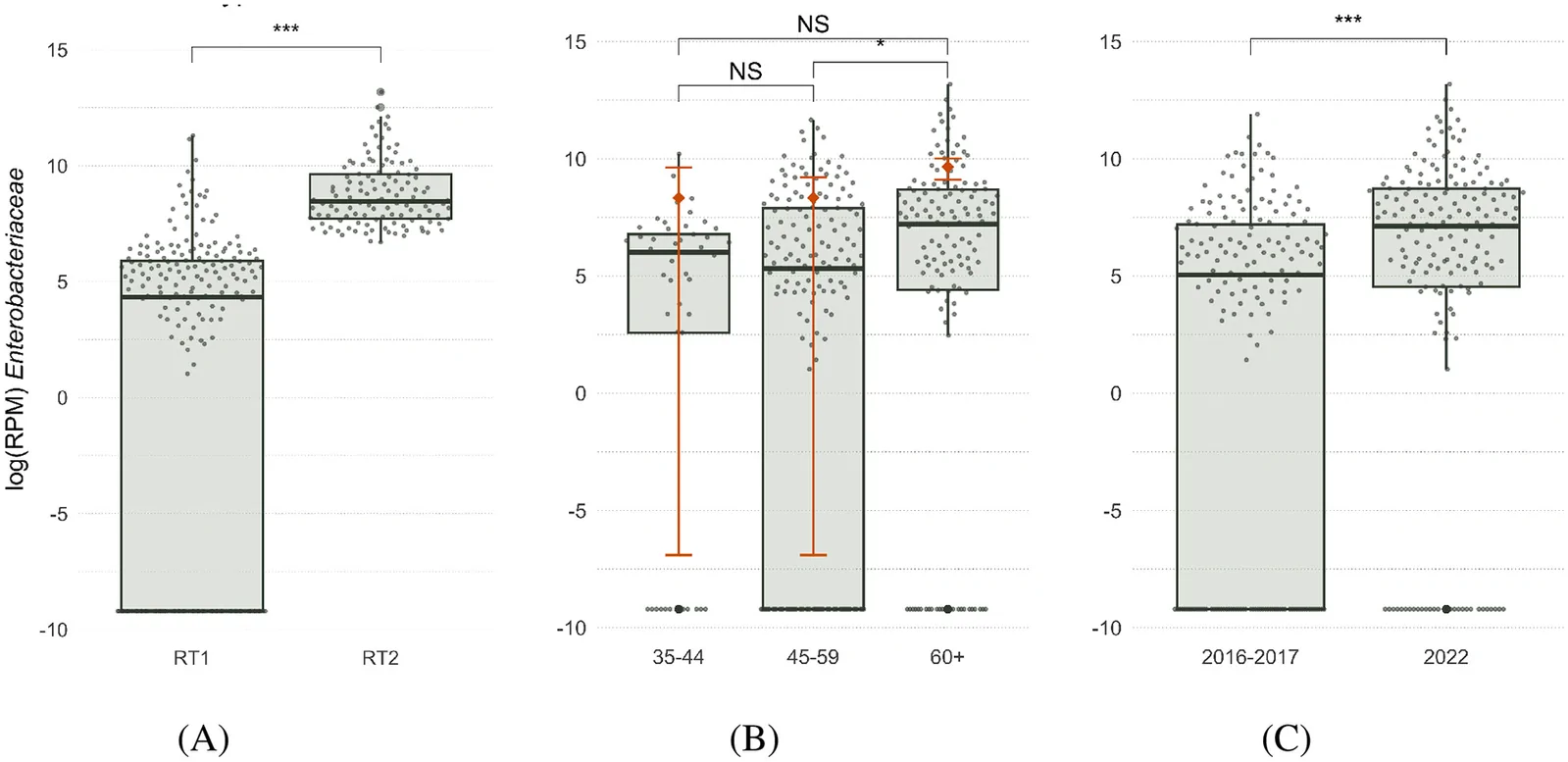
*Enterobacteriaceae* relative abundances (log-transformed, pseudocounts added) in relation to **(A)** resistotypes, **(B)** age groups, and **(C)** time period. For **(B)** orange points and error bars orange points and error bars represent estimated marginal means with 95% confidence interval from LMM. Significance levels for **(B)** are based on pairwise comparisons with Bonferroni correction. For **(A, C)** significance levels are based on Mann–Whitney *U*-test. *** – *p*-value < 0.001, * – *p*-value < 0.05, NS (not significant) – *p*-value > 0.05. Dots represent individual samples (*n* = 350).

## Discussion

Paired stool samples were collected 5 years apart to evaluate the potential impact of the COVID-19 pandemic on the gut resistome. This study was conducted using pre-existing stool samples collected in 2016-2017 during the KYH study conducted in Arkhangelsk, Russia (Cook et al., 2018), and a new set of samples collected in 2022. Participants of the 2016–2017 KYH study were re-contacted in 2022, and follow-up stool samples and registry-based data on their COVID-19 history were collected. While the 2022 samples were previously analyzed using targeted PCR in a study reporting associations between beta-lactam resistance genes and COVID-19 severity (Krieger et al., 2023), the current work employs shotgun metagenomic sequencing to comprehensively characterize longitudinal resistome dynamics across the two time points. While we observed no significant association between COVID-19 hospitalization history and resistome profiles, we identified changes in the resistome over a given 5-year period that we report below.

Among all ARGs identified in our data, aminoglycoside resistance genes exhibited the most notable prevalence increase. Specifically, *aph3, aph2*, and *ant9* were detected in less than 50% of samples during the 2016-2017 study period, but increased significantly by 2022, reaching up to 79% of samples (Figure 3). Another aminoglycoside resistance gene, *ant6*, maintained high prevalence (98% in 2022). These genes encode aminoglycoside-modifying enzymes (AMEs) that inactivate antibiotics through adenylation or phosphorylation, preventing ribosomal binding, and are frequently found on mobile genetic elements (plasmids, transposons, integrons, ISs), which facilitates horizontal gene transfer (Ramirez and Tolmasky, 2010; Garneau-Tsodikova and Labby, 2016). The persistent detection of aminoglycoside adenyltransferase *ant6* (*aadE*) aligns with its known widespread occurrence in human gut metagenomes (Feng et al., 2018). Historically, *ant6* was reported on *Tn*5405 transposon in *Staphylococcus aureus* (Derbise et al., 1996) and *Enterococcus faecium* (Werner et al., 2003), and later in *Streptococcus* (Cerdá et al., 2007). Aminoglycoside phosphotransferase *aph2* genes commonly occur in *Enterococcus* clinical isolates (Chen et al., 2006; Mahbub Alam et al., 2005), but were also detected in *Campylobacter* genomes (Yao et al., 2017). The aminoglycoside nucleotidyltransferase *ant9* (*aad9*) is frequently found in plasmids and transposons of *S. aureus, Enterococcus*, and other species and genera (Mahbub Alam et al., 2005; Wendlandt et al., 2013). Given the limited clinical use of aminoglycosides due to their toxicity, the drastic increase in aminoglycoside resistance gene prevalence is surprising. This phenomenon could potentially be explained by co-selection with other resistance determinants. The observed increase in prevalence, coupled with their frequent association with mobile genetic elements, suggests these ARGs may exist in a broader genomic context, potentially spreading among commensal gut species through horizontal gene transfer (HGT).

Three “core” macrolide resistance genes were detected in our data, namely, 23S rRNA methyltransferases *ermB, ermF*, and *ermG*. *ErmB* gene was the most prevalent, reaching 99% prevalence in 2022 (Figure 3). This gene belongs to multiple hosts and spreads via HGT, primarily within Firmicutes (Hu et al., 2016). This finding aligns with our recent study of a cohort sampled in 2021 in Moscow (Starikova et al., 2024), where *ermB* and *ermF* occurred in 95 and 87% of samples, respectively. Another macrolide resistance gene, *ermG*, increased in prevalence sharply from 33% to 53%. This gene wasn't previously reported as belonging to “core” human gut resistome, and such a notable increase in its prevalence is thereby concerning. The rise in prevalence observed for both *ermB* and *ermG* genes over a 5-year period may be partly explained by the use of macrolides during the early stage of COVID-19 pandemic (Krieger et al., 2023). Another possible explanation could be age-related antibiotic exposure, which we discuss below.

The simultaneous increase in prevalence of aminoglycoside and macrolide resistance genes observed in our study (Figure 3) suggests co-selection, possibly due to their genetic linkage. In Russia, azithromycin was widely prescribed at the beginning of the COVID-19 pandemic as part of official clinical recommendations (Krieger et al., 2023). This may have selected for macrolide resistance genes (*ermB*,*ermG*) as primary “drivers”, with linked aminoglycoside resistance genes co-selected as “passengers”, e.g. within mobile elements such as integrons. Physical linkage between *ermB* and the *aadE(ant6)–sat–aph3* gene cluster has been previously reported in gut commensal bacteria (Werner et al., 2003).

Several tetracycline resistance genes (*tetO, tetW, tetQ*, and *tet32*) encoding ribosomal protection proteins were near ubiquitous (99%–100% of samples) in both study periods (Figure 3). These genes are found in a wide range of bacterial genomes, both Gram-positive and Gram-negative (Connell et al., 2003; Chopra and Roberts, 2001), sometimes on mobile genetic elements (LeBlanc et al., 1988; He et al., 2025). Their high prevalence in our dataset aligns with previous reports showing that tetracycline resistance genes are among the most abundant and prevalent ARGs in human gut metagenomes across diverse populations (Qiu et al., 2020; Pal et al., 2016; Hu et al., 2013). However, this contrasts with relatively low tetracycline consumption, as noted in an article by Feng et al. evaluating ARG occurrence in 180 individuals from 11 different countries (Feng et al., 2018). The researchers suggested that the dominance of tetracycline resistance genes in the human gut may be due to their functions beyond antibiotic resistance, such as signaling or hydrophobic protein transport (Feng et al., 2018; Dantas and Sommer, 2012). Another possible explanation for the high prevalence of tetracycline resistance genes can be attributed to the use of tetracyclines in livestock, where they are used for therapeutic purposes and as growth promoters (Qiu et al., 2020; Grace, 2015). Residual tetracyclines in animal-derived products can exert low-level selective pressure in the human gut, thus favoring bacteria harboring *tet* genes. Comparative resistome analyses further reveal that tetracycline resistance genes also dominate animal microbiomes, with tetracycline resistance accounting for over 90% of ARG content (Pal et al., 2016). This overlap suggests potential transmission routes including direct contact with livestock, consumption of animal products carrying resistant bacteria, or environmental exposure to water and soil contaminated with animal waste. Taken together, these factors could potentially explain the near-ubiquitous presence of *tet* genes in human gut microbiomes despite declining clinical tetracycline usage.

Among the “non-core” ARGs identified in our study, several genes significantly increased their prevalence, mostly efflux pump genes belonging to the *Enterobacteriaceae* family (Supplementary Table 5). The prevalence of specific aminoglycoside (*aac6, sat*) and tetracycline (*tetBP*) resistance genes also changed, with *aac6* showing a particularly significant increase in prevalence over a 5-year period, aligning with the overall increase in aminoglycoside resistance genes, while *tetBP* prevalence decreased. The observed increase in *aac6* gene prevalence may reflect its known association with mobile genetic elements such as integrons (Chopra and Roberts, 2001). The increased prevalence of streptothricin acetyltransferase (*sat*) is surprising, given the discontinuation of streptothricin use in clinical and agricultural practice due to its toxicity (Franck and Crofts, 2024). However, this pattern might be explained by the known linkage of the *sat* gene to the two other aminoglycoside resistance genes, *ant6* (*aadE*) and *aph3* (Werner et al., 2003; Guirado et al., 2022; Lysnyansky and Borovok, 2021). These results suggest the need for monitoring aminoglycoside and macrolide resistance genes to prevent their further dissemination.

We identified two resistotypes in our data, with resistotype RT2 showing significantly higher ARGs richness than resistotype RT1 (medians: 85 and 21, correspondingly; Mann–Whitney *U*-test *p*-value < 0.001; Figure 4). Our results align with a recent study that clustered human intestinal ARGs profiles into two resistotypes (Lee et al., 2023). Similar to our findings, the less common resistotype had higher overall ARG relative abundance, pre-dominantly associated with species-specific genes within the Proteobacteria phylum, including *Enterobacteriaceae*. In our study, the less common resistotype 2 was also characterized by an increased relative abundance of multidrug resistance efflux pump and beta-lactamase genes, as shown by two differential abundance methods, DESeq2 and MaAsLin (*p*-values < 0.05; Supplementary Figures 10, 11). Most of the genes identified as RT2-specific correlated with *Enterobacteriaceae* family relative abundance (Supplementary Figures 8, 16-18).

We found that resistotypes were strongly associated with age (Mann–Whitney *U*-test *p*-value < 0.001; Figure 7B). Participants with RT2 were, on average, 5 years older than those with RT1, and the proportion of samples assigned to RT2 gradually increased with participant age (Figure 7A). This aligns with our observation that older participants had higher ARGs richness and ARG load (LMM pairwise comparisons, *p*-value < 0.05; Figure 6), which is supported by findings from the previous research (Wu et al., 2021; Tavella et al., 2021). One of the studies reported the accumulation of specific ARGs with age, particularly genes associated with efflux pumps (Tavella et al., 2021), which is consistent with our results: resistotype 2 that is more common in the 60+ age group had a higher relative abundance of efflux pump genes (Supplementary Figures 10, 11). Aging of the participants might also explain the increased number of detectable ARGs in 2022 (Supplementary Figure 5).

The link between the gut resistome and age was further underscored by the uneven changes in resistome composition across different age groups over time. The 61-65 and 41-45 age groups showed more pronounced changes over 5 years. This pattern aligns with the concept of the two turning points in the aging process that were described in a recent study based on multi-omics data (Shen et al., 2024): these critical points occur at approximately 44 and 60 years of age and are associated with the onset of certain age-related diseases.

Age-related changes in the gut microbiome are well documented and include a decline in microbial diversity (Gao et al., 2026), reduced abundance of beneficial commensals, and an expansion of opportunistic taxa such as *Enterobacteriaceae*, which are frequent carriers of mobile antibiotic resistance genes. Such alterations in the intestinal environment favor its colonization by resistant strains. Furthermore, in older adults, a higher burden of chronic disease leads to more frequent healthcare use and increased susceptibility to infections, resulting in greater antibiotic exposure, which might explain the observed accumulation of ARGs (Theodorakis et al., 2024).

One of the two resistotypes identified in our data, RT2, was associated with an increased *Enterobacteriaceae* relative abundance (Mann–Whitney *U*-test *p*-value < 0.001; Figure 8A). We also found that the participants aged 60 and older had higher *Enterobacteriaceae* relative abundance than the 45-59 age group (LMM pairwise comparisons, *p*-value < 0.05; Figure 7). This observation aligns with the previous studies that showed age-related increase in *Enterobacteriaceae*, in the gut microbiota, reflecting shifts in gut function associated with aging (Biagi et al., 2010; Rampelli et al., 2013).

Longitudinal studies of resistomes are critical to track their adaptation to selective pressures. Previous analyses that included 12 healthy individuals demonstrated that resistotypes can undergo short-term, reversible shifts in response to antibiotic treatment (Lee et al., 2023). In our study, we analyzed a larger cohort of participants over a longer time period and found that about one-third of the participants changed their resistotype between 5 years Figure 5B. While no significant associations were detected between resistotype dynamics and available participants' metadata, the median age of the participants transitioning from RT1 to RT2 was 4 years higher than those retaining RT1 (55 and 51 years, correspondingly; Supplementary Figure 16). This observation aligns with the higher prevalence of RT2 in older participants Figure 7C, suggesting that aging may contribute to resistotype shifts. However, the observed age difference was not statistically significant, which may reflect the small magnitude of the difference combined with reduced statistical power due to sample size constraints.

The observed shift in resistotypes may also reflect increased antibiotic use during the COVID-19 pandemic. Our study found that participants with a history of COVID-19 hospitalization had a higher mean ARG richness than non-hospitalized participants (45 and 36 genes, correspondingly; Supplementary Figure 12e). Although this difference did not reach statistical significance (*p*-value = 0.06), possibly due to small sample size, the trend suggests a potential association between severe COVID-19 and increased antimicrobial resistance due to antibiotic treatment and horizontal gene transfer from hospital-associated microbiota during prolonged hospitalizations. The increase in the prevalence of macrolide resistance genes observed in our study (Figure 3) may be related to the use of azithromycin during the COVID-19 pandemic: this antibiotic was one of the most frequently prescribed in Russia for its immunomodulatory effects in the early stages of COVID-19 (Rachina et al., 2022).

Our study revealed no sex-specific differences in resistome composition (Supplementary Figures 12a, 13a), contrasting with a Dutch populational study (Sinha et al., 2019). That study reported higher ARGs richness in females compared to males, with the most notable difference observed for the lincosamide resistance genes. The authors attributed these results to national sex differences in antibiotic consumption. The discrepancy between the Dutch and the Russian cohorts may reflect country-specific antibiotic prescription policies.

A recent meta-analysis showed increased ARGs richness in the gut microbiome associated with obesity (Hu et al., 2024). In our study, no differences in ARGs richness was found between BMI groups (Supplementary Figure 12b). However, we observed significantly higher ARG load in obese (BMI > 30 kg/m^2^) participants as compared to overweight (25 < BMI ≤ 30 kg/m^2^) participants (Supplementary Figure 13b). Additionally, individuals with higher BMI exhibited greater Bray-Curtis dissimilarity in their paired samples (Supplementary Figure 14a), suggesting more pronounced temporal changes in ARGs composition compared to those with a lower BMI. This may reflect obesity-related metabolic and inflammatory changes, which may lead to a less stable gut microbiome, resulting in greater shifts in ARGs profiles over time (Fan and Pedersen, 2021). Individuals with higher BMI may have poorer diet quality, increased susceptibility to infection, and greater use of antibiotics, which could contribute to changes in ARGs composition (Jacob et al., 2022; Falagas and Kompoti, 2006).

This study has several limitations, with major ones being the 5-year gap between the sampling points and the lack of antibiotic exposure data during this interval. This study design doesn't allow us to analyse interim resistome dynamics and omits an important factor influencing resistome. Moreover, without individual-level antibiotic use data, the observed age-related resistome patterns cannot be attributed to aging alone. In studies with long-term community-based design, obtaining detailed antibiotic intake history is challenging in the absence of continuous clinical monitoring. The analysis of age-related effects is also constrained by the available cohort, which did not include participants under 35 years of age. Furthermore, the division of the cohort into subgroups resulted in small sample sizes for some categories, potentially limiting statistical power. Finally, our analysis relied on a curated subset of the MEGARes database, which is biased toward well-characterized species, and ARGs belonging to commensal bacteria are underrepresented. Future databases integrating resistance-associated sequences from under-represented taxa could significantly improve the accuracy and scope of resistome analyses.

Overall, our study offers a valuable longitudinal perspective on gut resistome dynamics, capturing changes over a 5-year interval in a defined population. To date, this is the first study to link human gut resistotypes with chronological age, and the first study to document a substantial increase in prevalence of aminoglycoside and macrolide resistance genes during COVID era.

## Conclusions

Our findings demonstrate the changes of the intestinal resistome of the residents of Arkhangelsk, Northwestern Russia, over a 5-year period. Although the study period included the pandemic, no significant associations were found between the characteristics of the resistome and the COVID-19 history. We found that both overall ARGs richness and ARG load increased over 5 years, as well as the prevalence of certain genes, with the most pronounced prevalence changes shown for ARGs associated with resistance to aminoglycosides and macrolides. We identified two distinct resistotypes within our samples, with one of the resistotypes (RT2) being associated with a higher participants' chronological age and higher *Enterobacteriaceae* relative abundance. About one-third of the participants who had RT1 at the baseline shifted to RT2 within a 5-year interval. This effect could be potentially explained by the aging of the participants, although we couldn't link transition type to the participants' age with statistical significance, possibly due to the limited number of the samples belonging to a less frequent RT2. We also found that temporal changes in resistomes occurred unevenly across baseline age groups, with 61-65 and 41-45 age groups showing the most pronounced changes. To our knowledge, this is the first study to link resistotypes with age; however, larger population-based studies are required to confirm these associations and explore the mechanisms driving age-related trends in resistome dynamics.

## Data Availability

The sequencing reads have been deposited in the NCBI BioProject database under accession number PRJNA1247984 (https://www.ncbi.nlm.nih.gov/bioproject/PRJNA1247984). The data-processing and resistome-profiling pipeline is available on GitHub at https://github.com/bobeobibo/ResistoViper. The R scripts used for data analysis and figure generation, along with the input data, are available on Zenodo at https://zenodo.org/records/21790852.
