# Supplementary material for: Gut microbiome resistotypes reflect chronological age and Enterobacteriaceae relative abundance
Source: Front Microbiol. 2026 Sep 30;17:1869983. doi: 10.3389/fmicb.2026.1869983 (PMC13661311; doi:10.3389/fmicb.2026.1869983)
Supplement: Supplementary file 1 [file Data_Sheet_1.pdf]

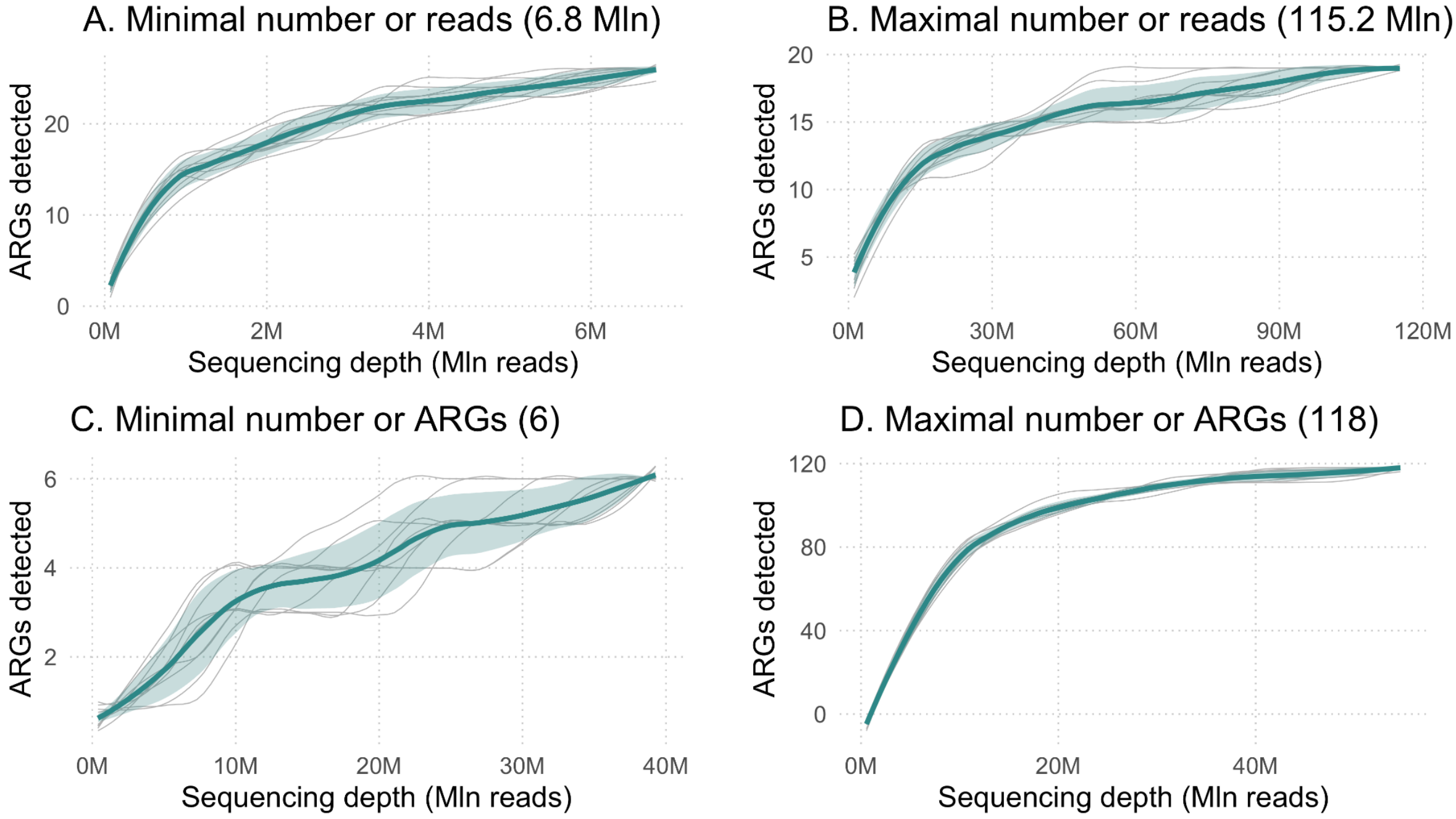


**Supplementary Figure 1.** Saturation of ARGs richness with increasing sequencing depth. Rarefaction was performed in 100 steps for samples with the **A**. minimal number of reads, **B**. maximal number of reads, **C**. minimal ARGs richness, and **D**. maximal ARGs richness. Grey curves represent 10 iterations per sample, smoothed using LOESS. The bold teal curve represents the smoothed mean of all iterations. The ladder-like pattern in **C**. is an expected artifact of rarefying a sample with a low number of ARGs (n=6). The ribbon indicates the standard deviation around the mean.

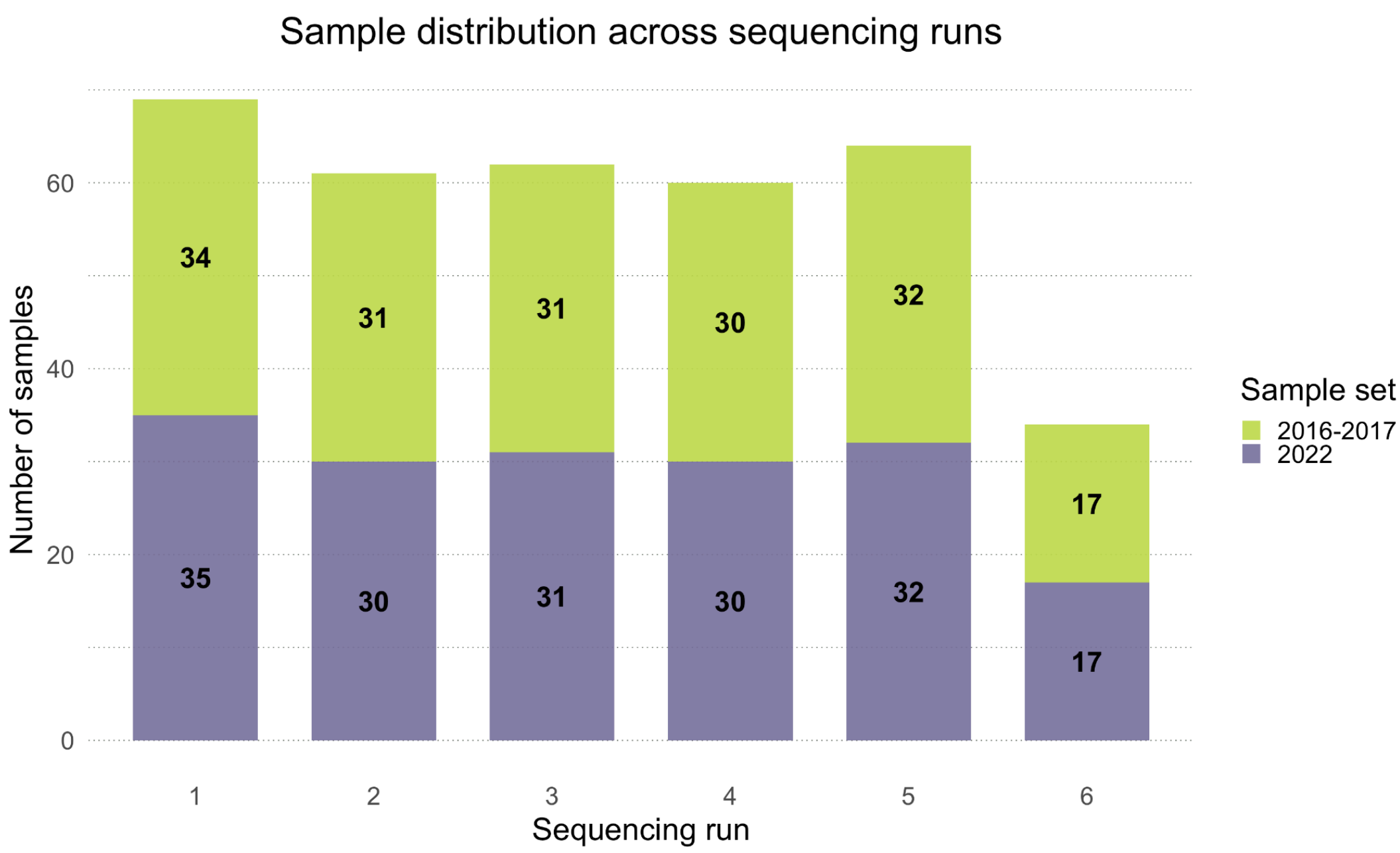


**Supplementary Figure 2.** Sample distribution across sequencing runs.

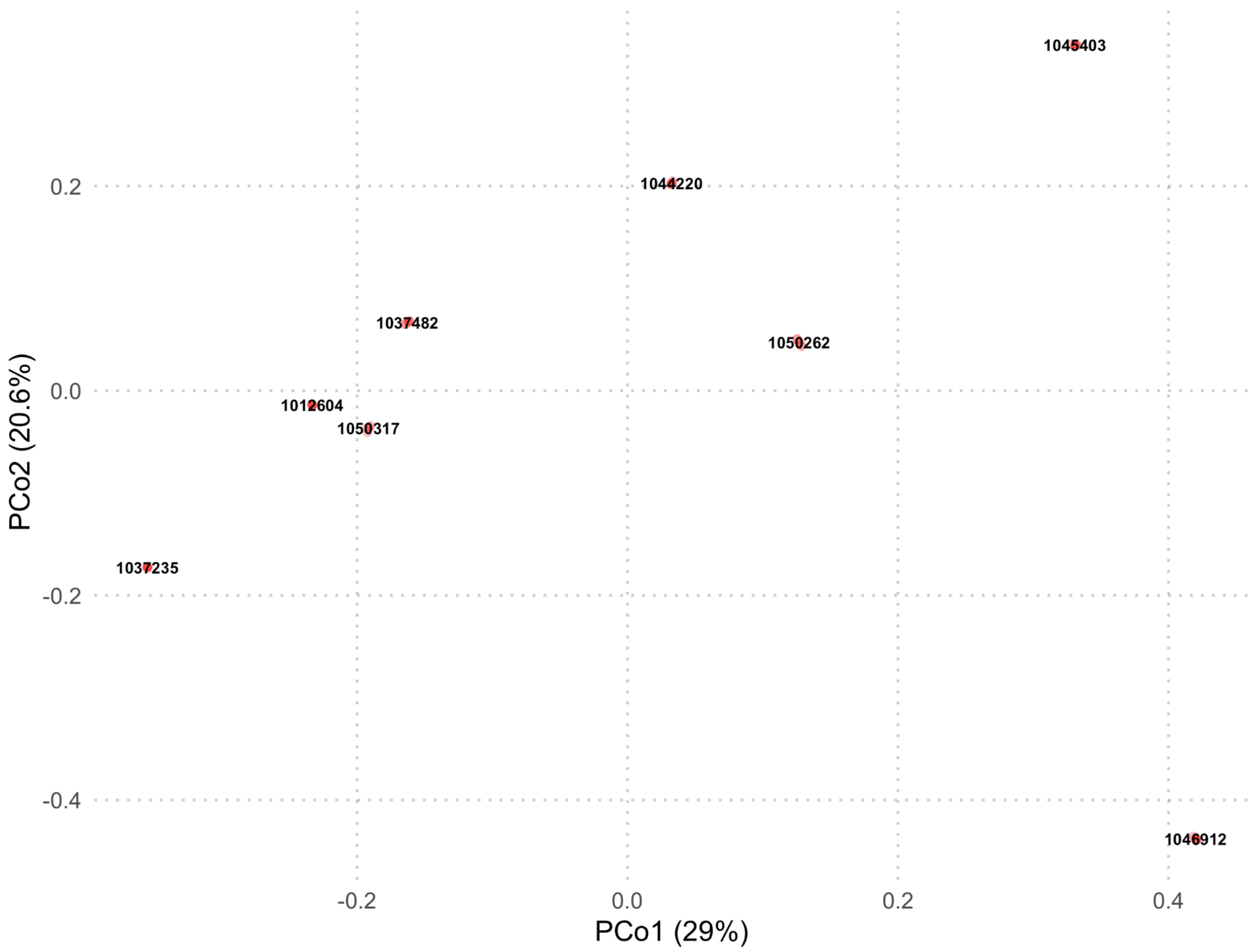


**Supplementary Figure 3.** PCoA ordination (Bray-Curtis index) based on genus-level taxonomical composition of technical repeats sequenced in separate batches (6 repeats per each of biological samples, 48 samples in total). Labels indicate biological sample ids.

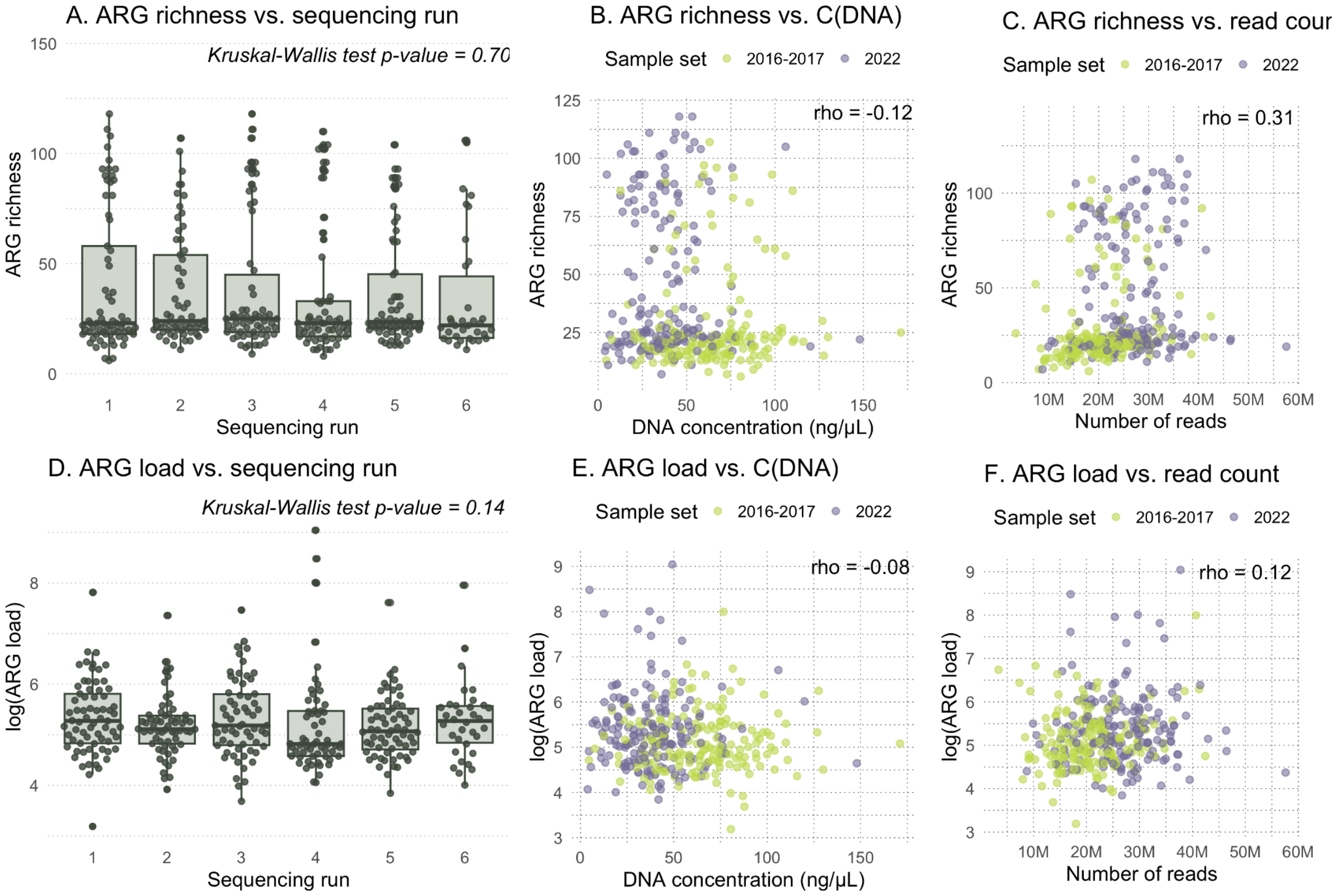


**Supplementary Figure 4.** ARG richness (**A**-**C**) and ARG load (**D**-**F**) metrics in relation to sequencing run, DNA concentration and read count. Spearman's correlation coefficients are shown on panes **B**, **C**, **E** and **F**.

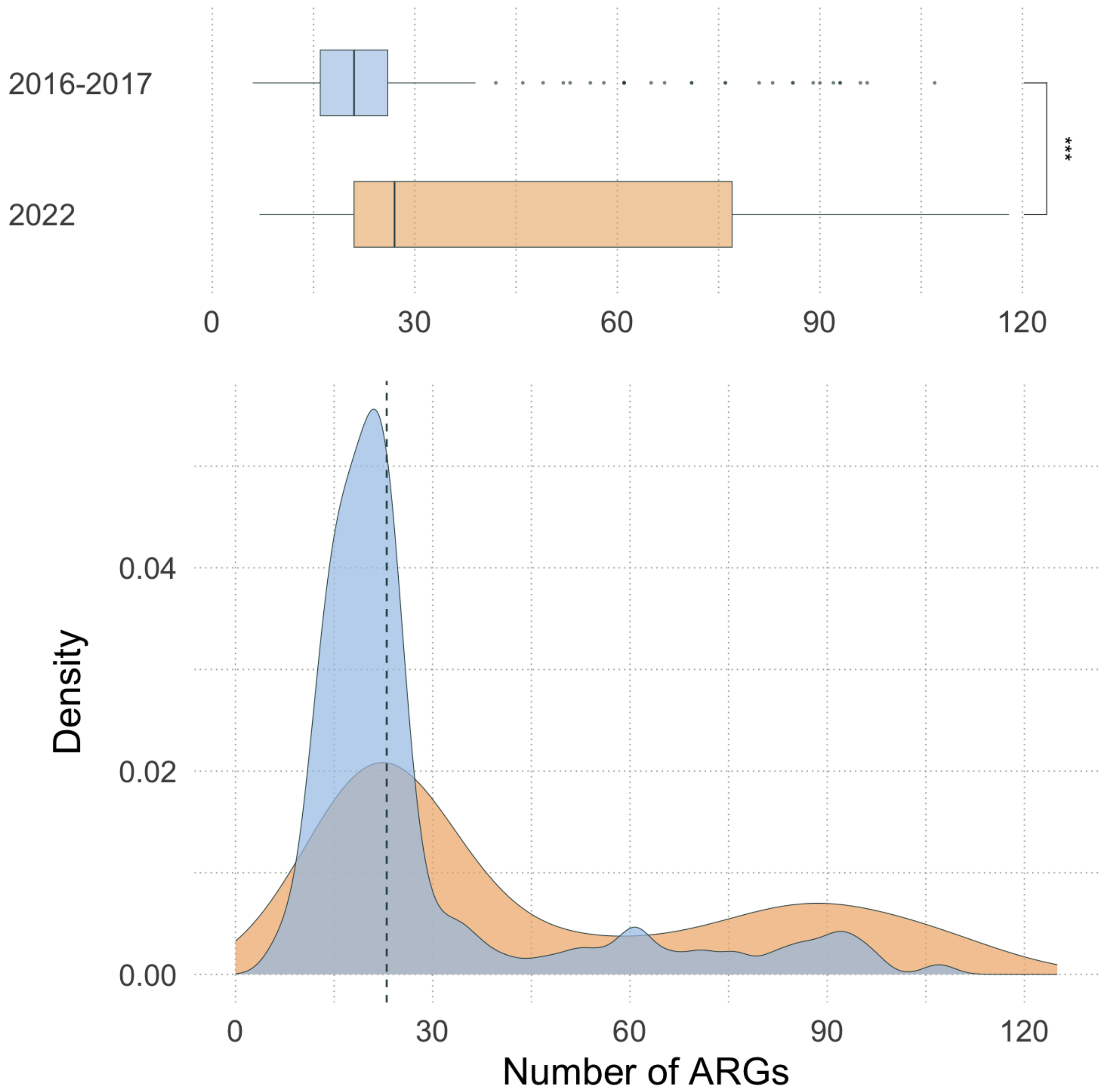


**Supplementary Figure 5.** A combined plot showing the distribution of the number of ARGs in each sample set (blue — 2016-2017, 175 samples; orange — 2022, 175 samples). *** — Wilcoxon signed-rank test p-value < 0.001. Vertical dashed line represents median value.

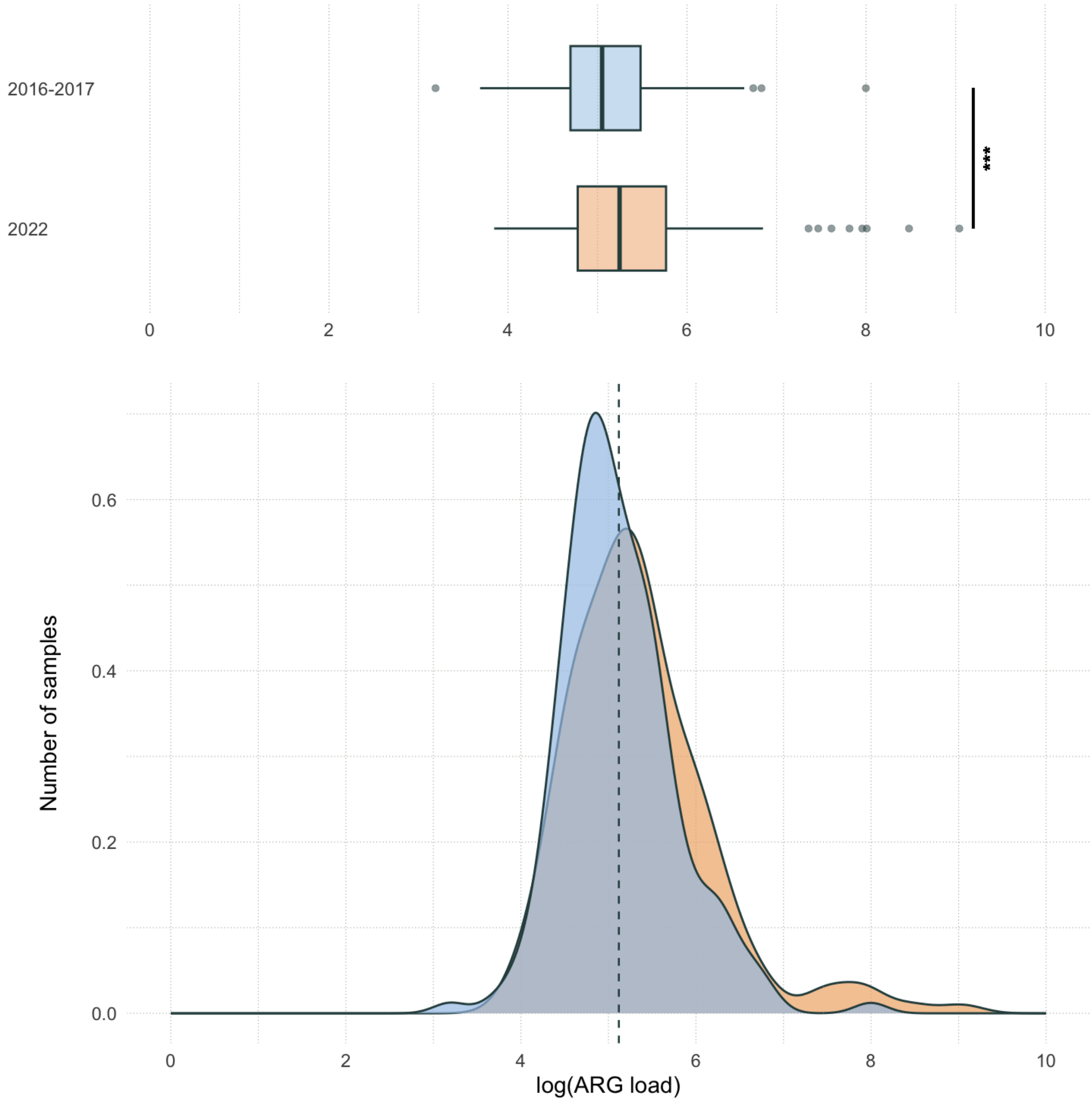


**Supplementary Figure 6.** A combined plot showing the distribution of the ARG load values in each sample set (blue — 2016-2017, 175 samples; orange — 2022, 175 samples). *** — Wilcoxon signed-rank test p-value < 0.001. Vertical dashed line represents median value.

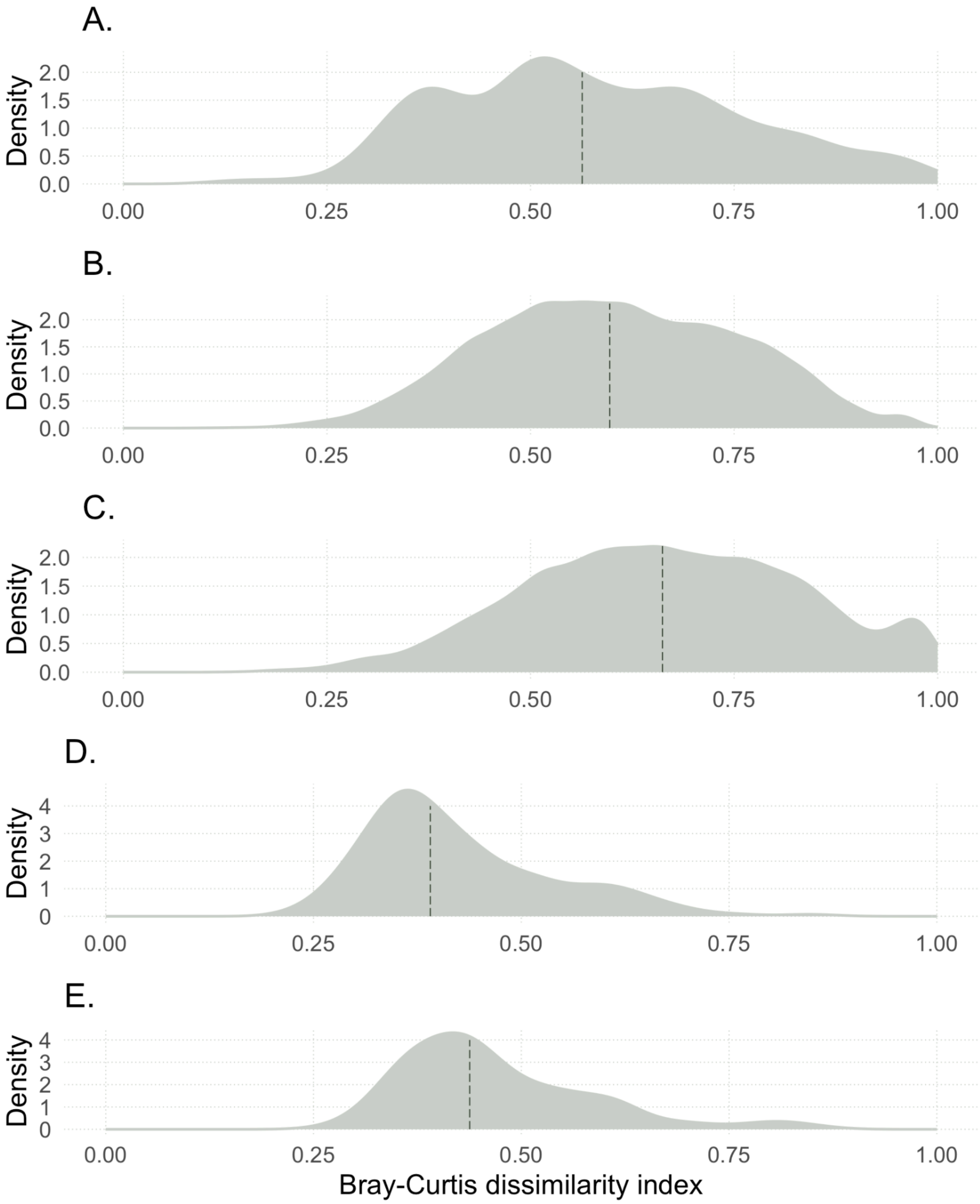


**Supplementary Figure 7.** Density plots showing distributions of Bray-Curtis indices: **A**. paired samples from the same individuals (2016-2017 vs. 2022, n = 175), **B**. all pairwise comparisons within 2016-2017 samples (n = 30450), **C**. all pairwise comparisons within 2022 samples (n = 30450), **D**. distances from 2016-2017 samples to their group centroids (PERMDISP, n = 175), **E**. distances from 2022 samples to their group centroids (PERMDISP, n = 175). Dashed lines represent median values.

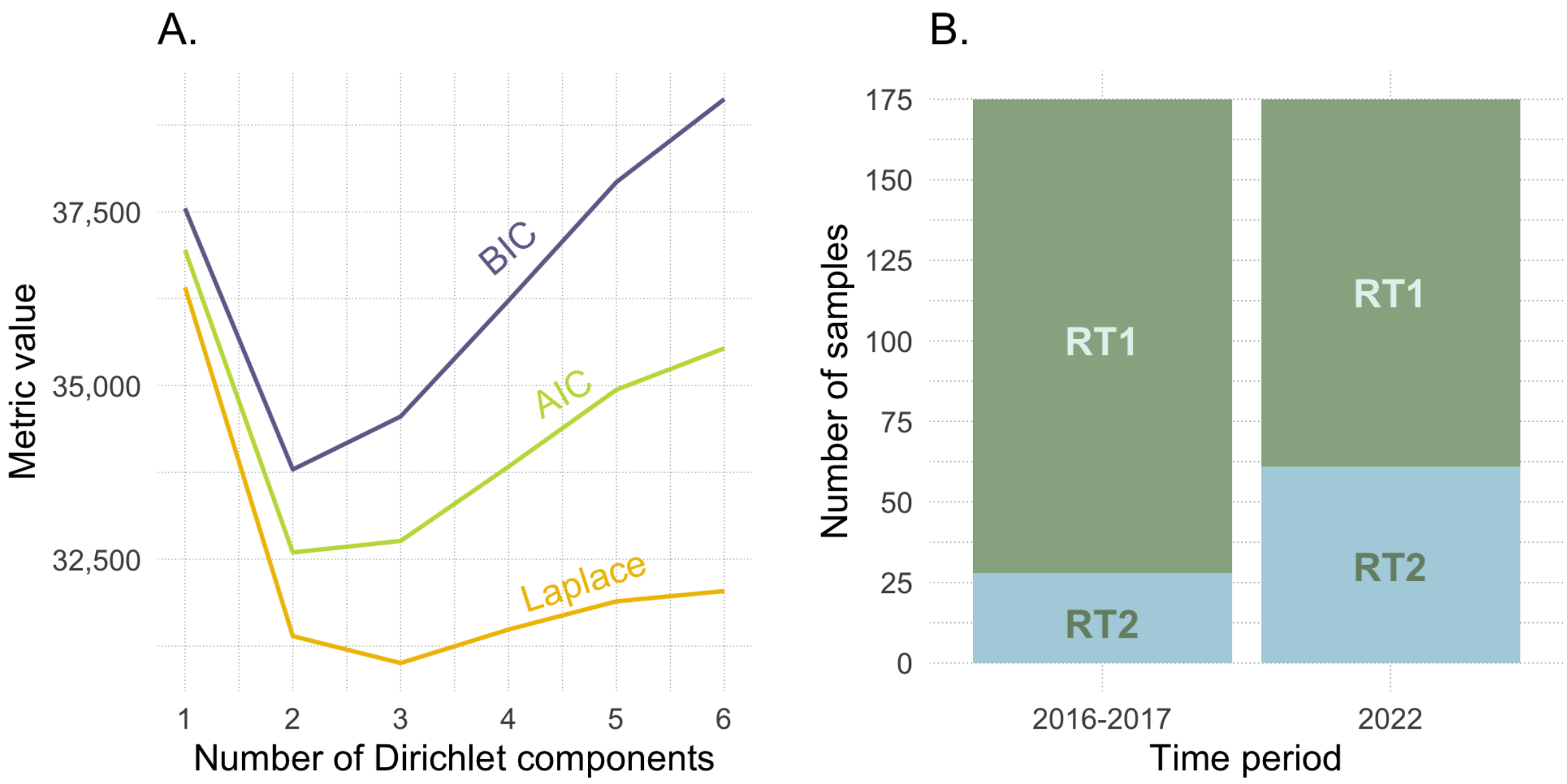


**Supplementary Figure 8. A.** Model fit evaluation for Dirichlet Multinomial Mixture Models across varying numbers of components performed based on RPKM values of all samples from both years. **D**. Resistotype proportions in each year. RT1 — resistotype 1, RT2 — resistotype 2.

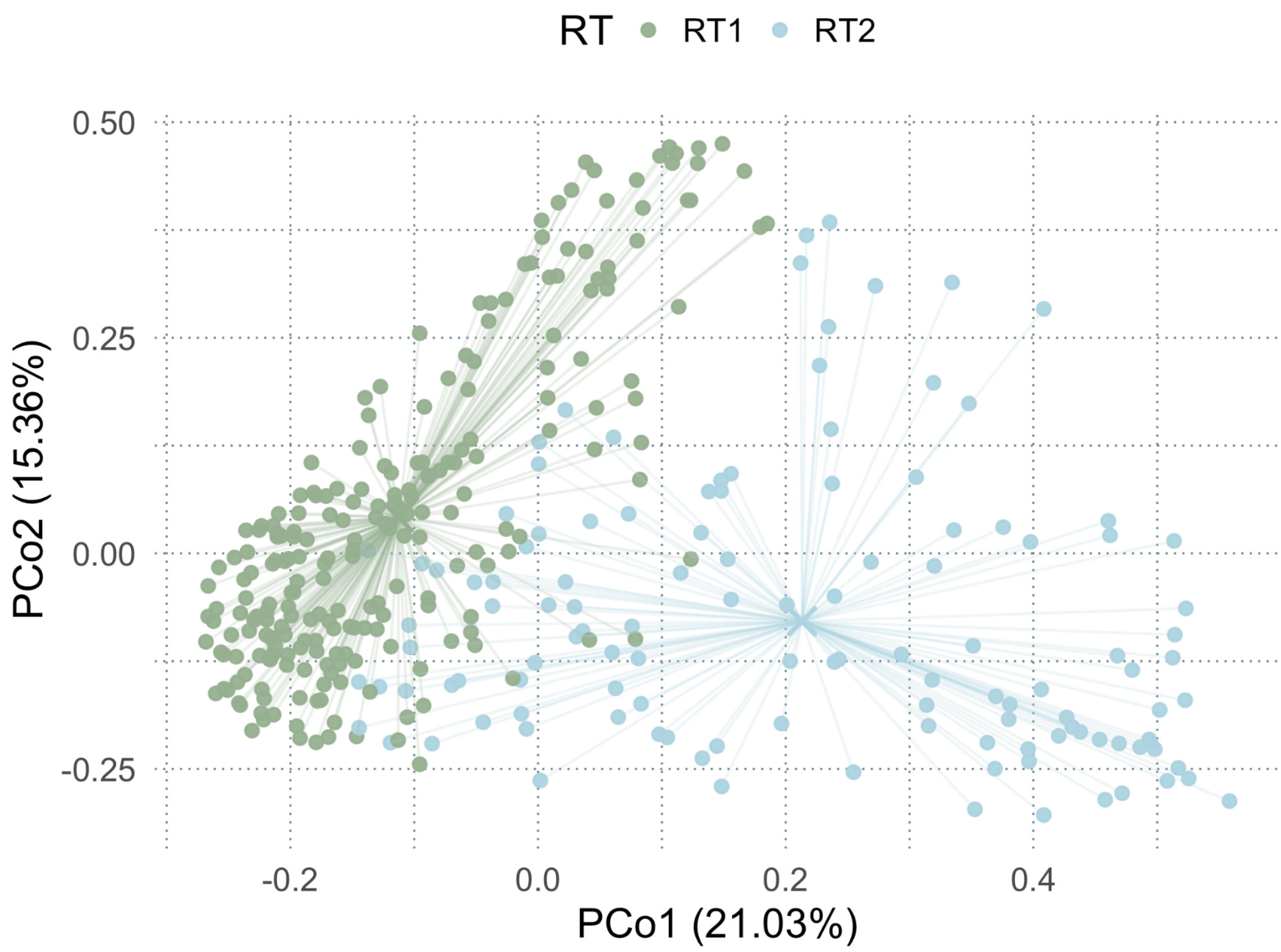


**Supplementary Figure 9.** PCoA plot of gut resistome profiles based on Bray-Curtis dissimilarity indices of all samples. Samples are colored by resistotype (RT) assignment.

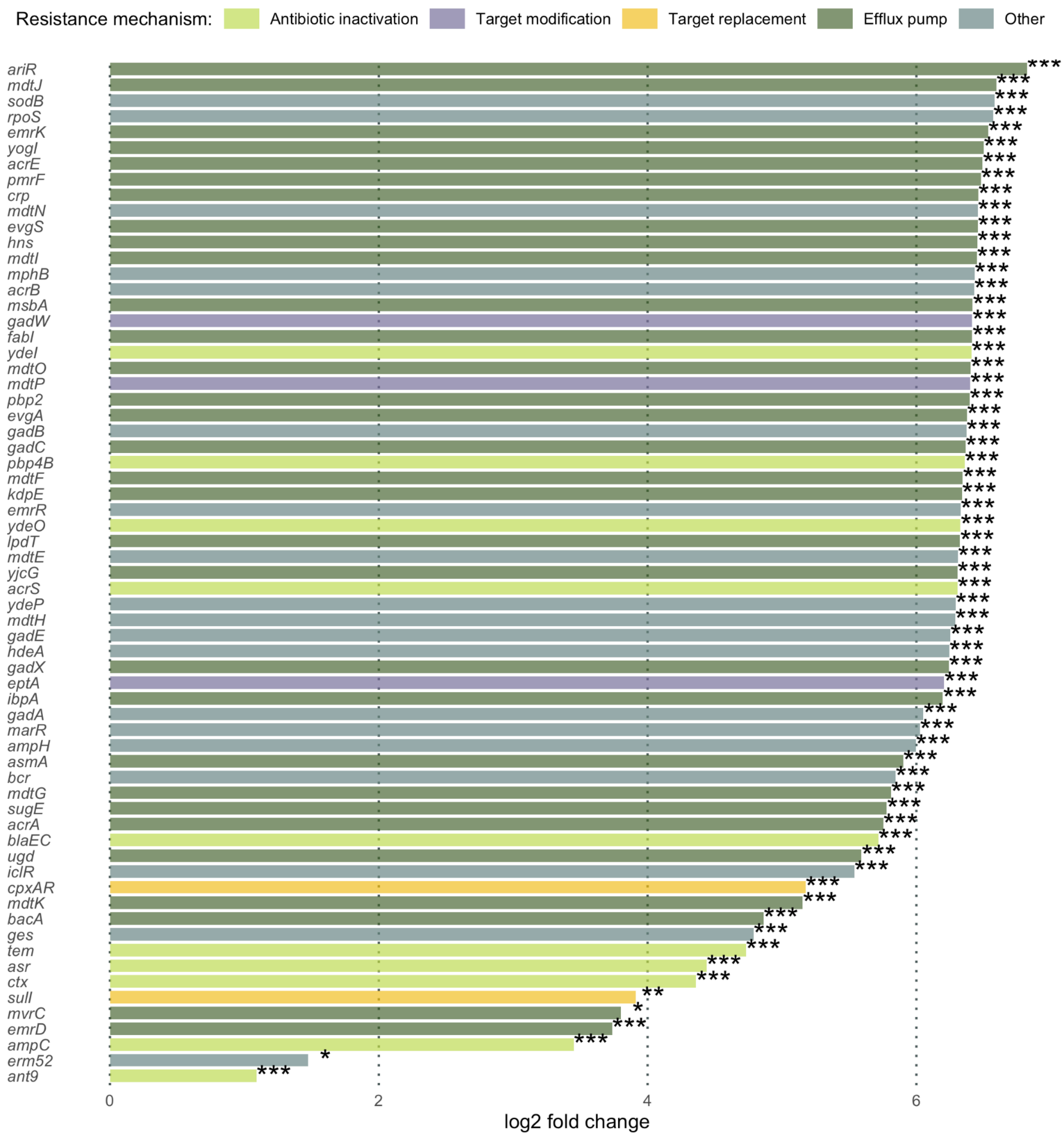


**Supplementary Figure 10.** Barplot illustrating log2 fold change of ARG read counts between resistotypes, obtained with DESeq2 method. All the bars correspond to ARGs overrepresented in RT2 (resistotype 2). *** — adjusted p-value < 0.001, ** — adjusted p-value < 0.010, * — adjusted p-value < 0.050.

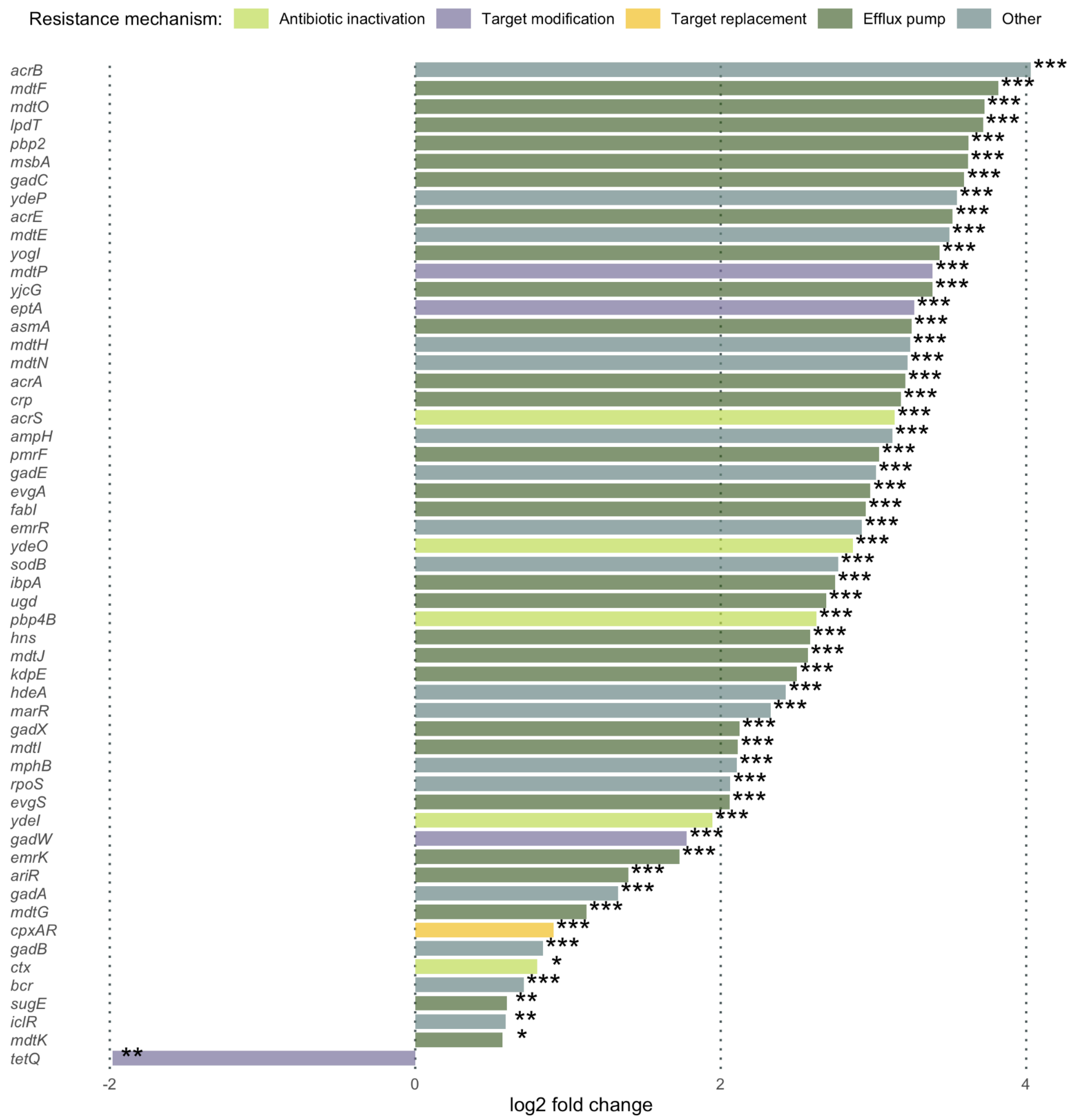


**Supplementary Figure 11.** Barplot illustrating log2 fold change of ARG read counts between resistotypes, obtained with MaAsLin3 method. All the bars correspond to ARGs overrepresented in RT2 (resistotype 2). *** — adjusted p-value < 0.001, ** — adjusted p-value < 0.010, * — adjusted p-value < 0.050.

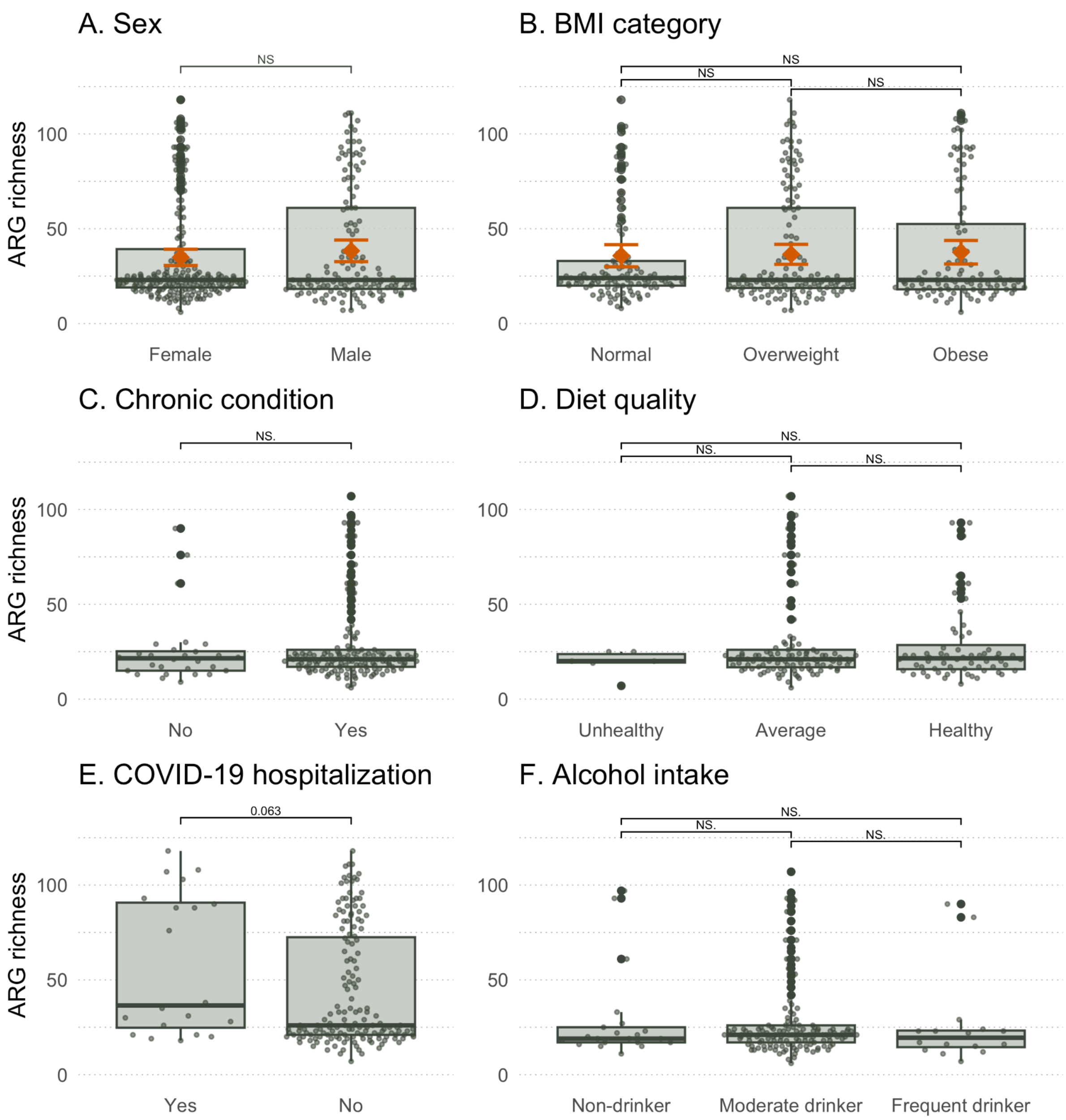


**Supplementary Figure 12.** ARGs richness in relation to **A**. sex (all samples considered, n = 350), **B**. BMI category (all samples considered, n = 350), **C**. the presence of a chronic condition (2016-2017 samples, n = 175), **D**. diet quality (2016-2017 samples, n = 175), **E**. COVID-19 hospitalization (2022 samples, n = 175), and **F**. the frequency of alcohol intake (2016-2017 samples, n = 175). For **A** and **B**, orange points and error bars represent estimated marginal means with 95% confidence interval from LMM (with year, sex, BMI, age group as fixed effects, and participant ID as random effect); significance levels are based on pairwise comparisons with Bonferroni correction. For **C-F**, significance levels are based on Mann-Whitney U test. NS — p-value >0.05. Dots represent individual samples.

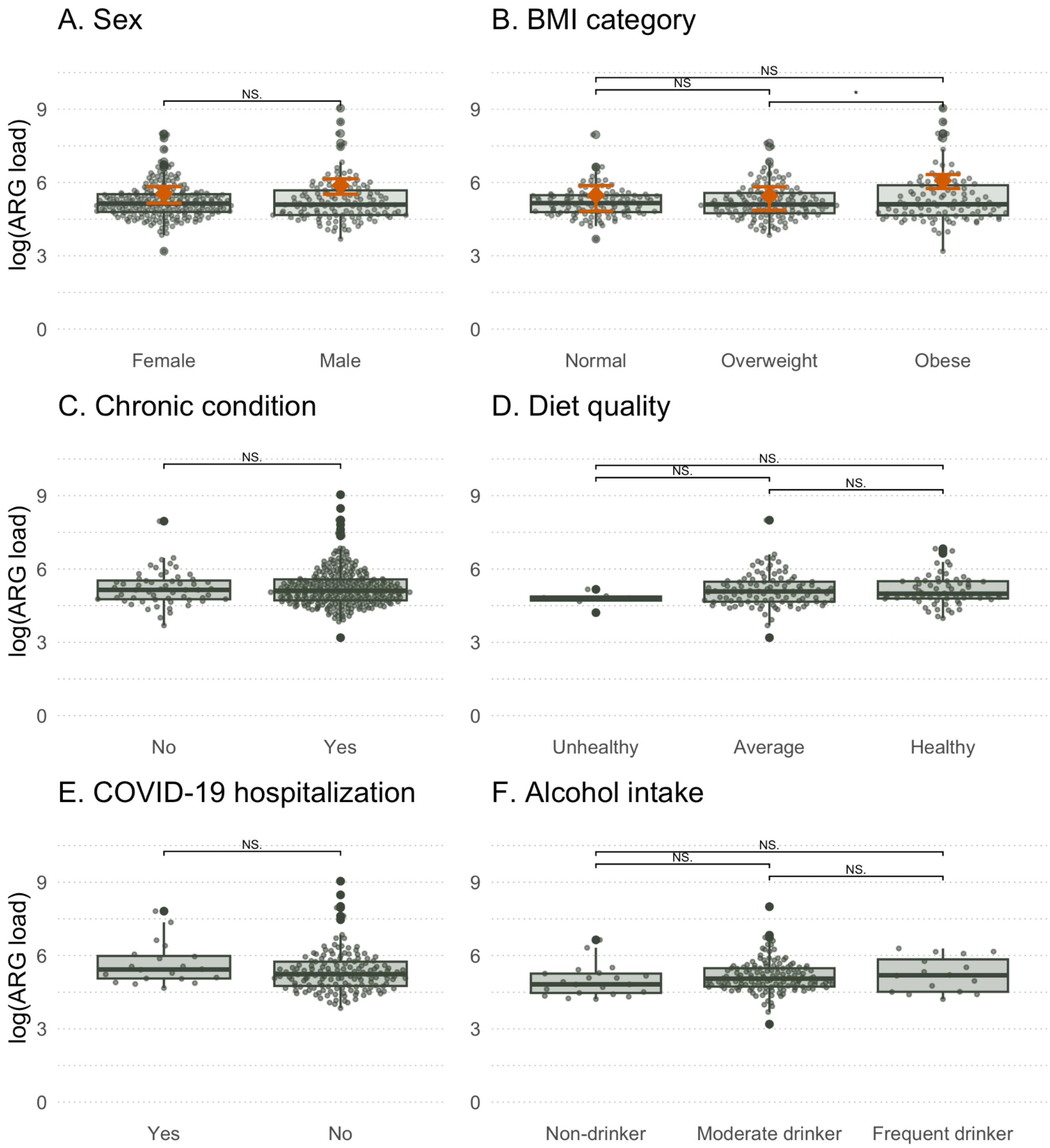


**Supplementary Figure 13.** ARG load values (log-transformed) in relation to **A**. sex (all samples considered, n = 350), **B**. BMI category (all samples considered, n = 350), **C**. the presence of a chronic condition (2016-2017 samples, n = 175), **D**. diet quality (2016-2017 samples, n = 175), **E**. COVID-19 hospitalization (2022 samples, n = 175), and **F**. the frequency of alcohol intake (2016-2017 samples, n = 175). For **A** and **B**, orange points and error bars represent estimated marginal means with 95% confidence interval from LMM (with year, sex, BMI, age group as fixed effects, and participant ID as random effect); significance levels are based on pairwise comparisons with Bonferroni correction. For **C-F**, significance levels are based on Mann-Whitney U test. * — p-value < 0.05; NS — p-value >0.050. Dots represent individual samples.

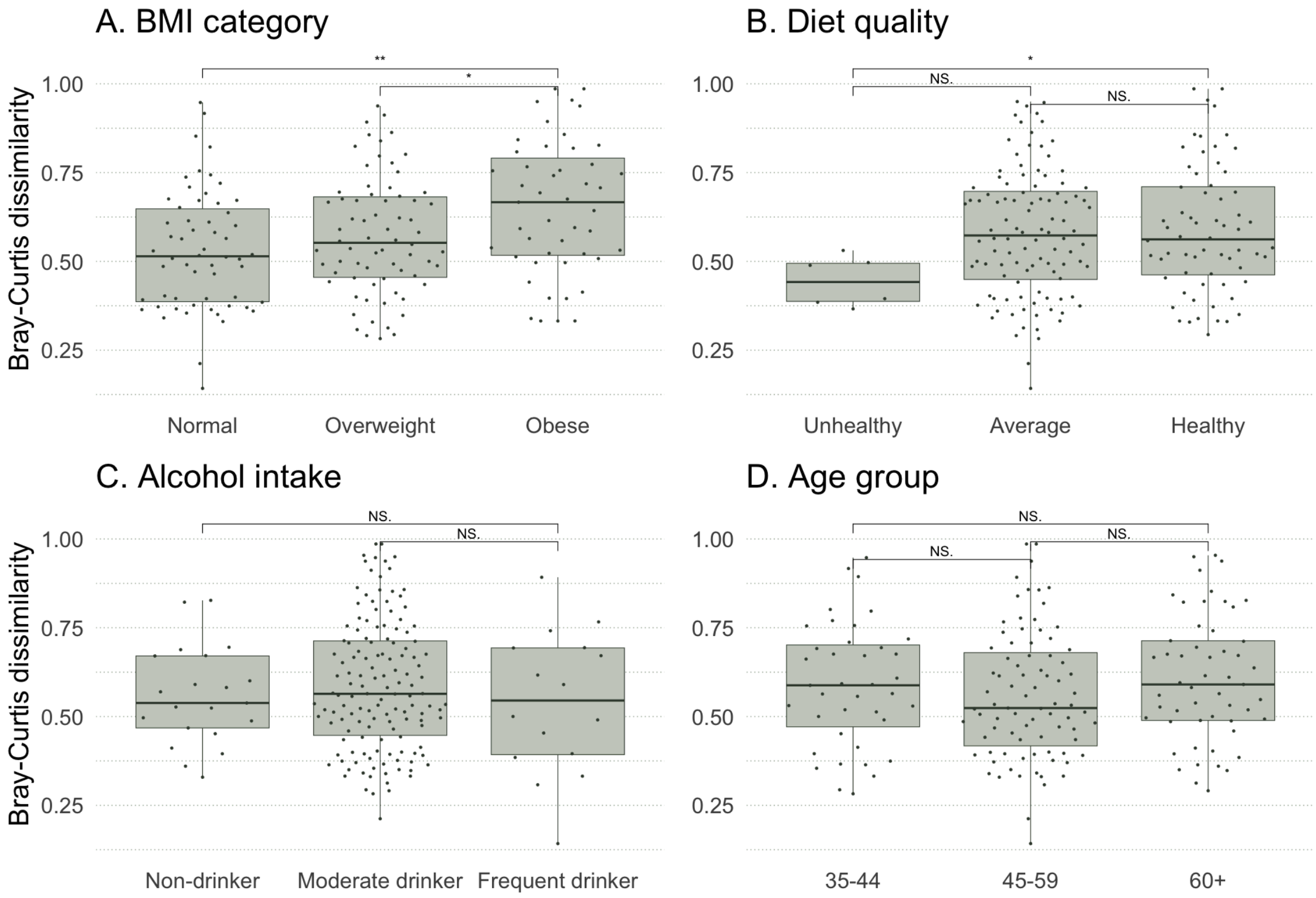


**Supplementary Figure 14.** Bray-Curtis dissimilarity indices between paired samples in relation to **A**. BMI (body mass index) group, **B**. diet quality, **C**. the frequency of alcohol intake, and **D**. age group. All the measurements were taken at baseline (2016-2017). * — Mann-Whitney U test p-value < 0.05, NS (not significant) — p-value >0.050. Dots represent individual samples (n = 175).

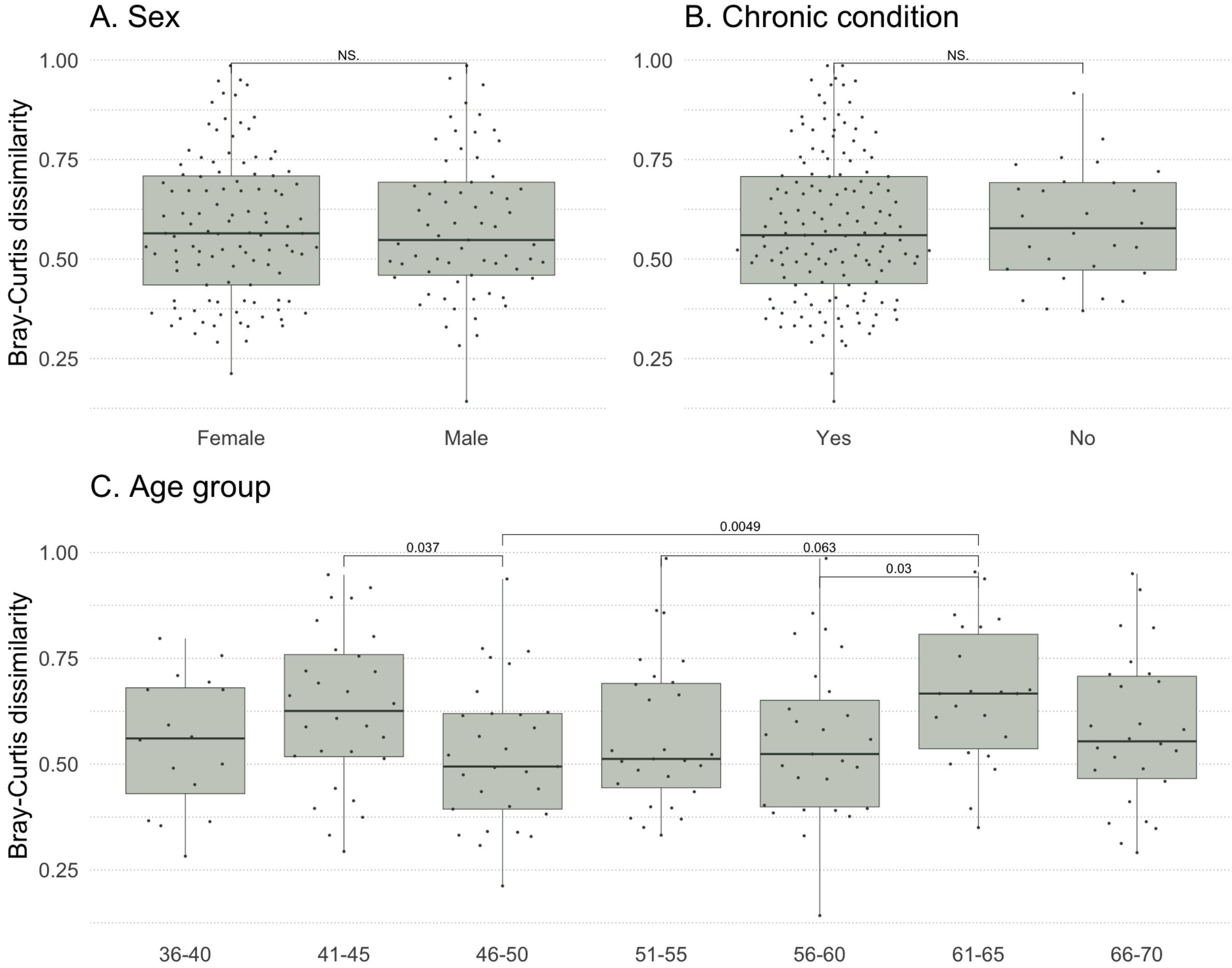


**Supplementary Figure 15.** Bray-Curtis dissimilarity indices between paired samples in relation to **A**. sex, **B**. the presence of a chronic condition, and **C**. age group. All the measurements were taken at baseline (2016-2017). Numbers above boxes illustrate Mann-Whitney U test p-values. NS (not significant) — Mann-Whitney U test p-value >0.050. Dots represent individual samples (n = 175).

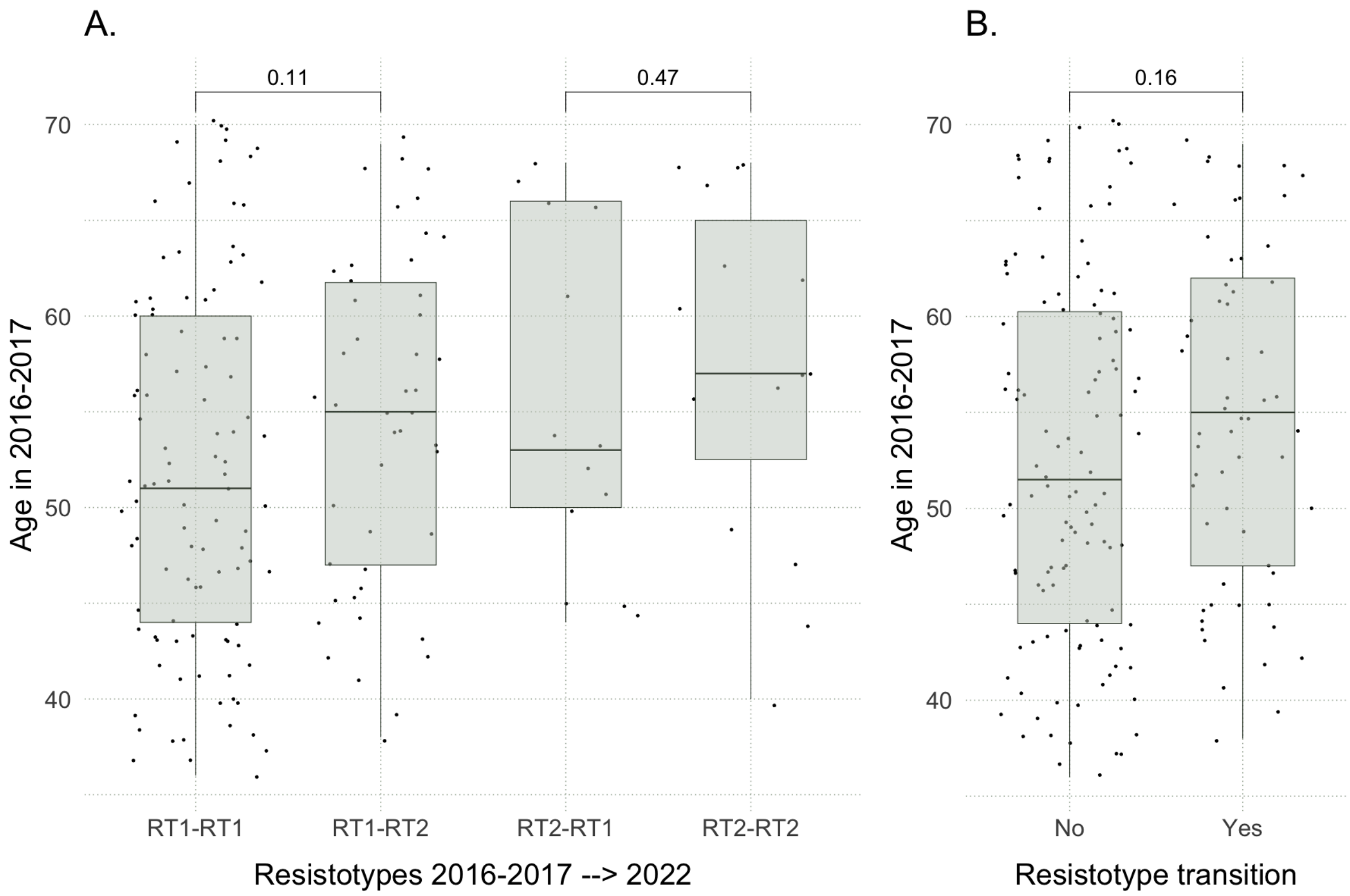


**Supplementary Figure 16.** Boxplots illustrating the changes in resistotypes between 2016-2017 and 2022 in relation to the participants' age at baseline. Numbers above the boxes illustrate Mann-Whitney U test p-values. Dots represent individual samples (n = 175).

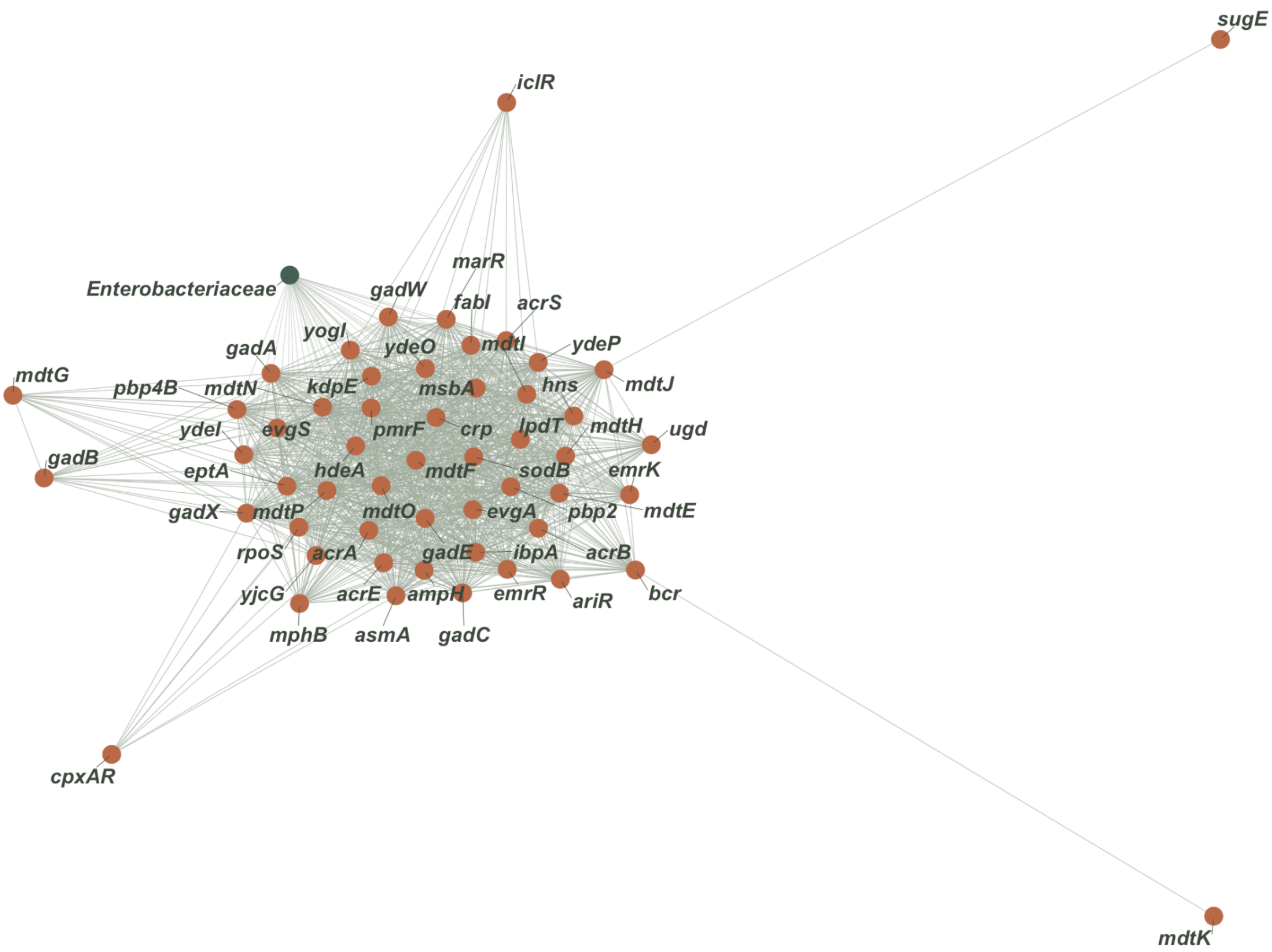


**Supplementary Figure 17.** Graph showing relative abundance correlations between RT2-specific ARGs and bacterial families. Only “gene—gene” and “gene—family” pairs with high (> 0.7) Spearman’s correlation coefficients are shown.

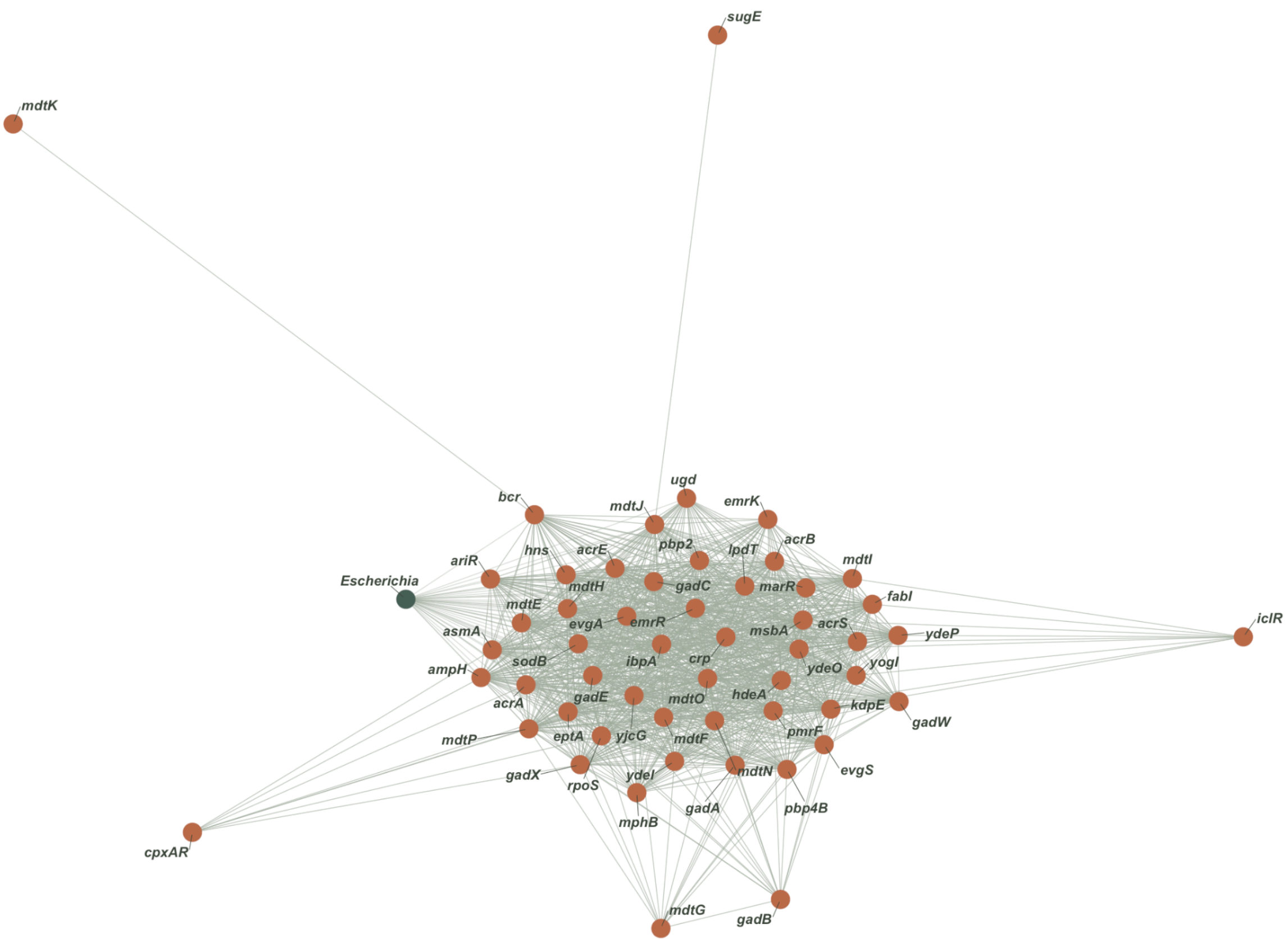


**Supplementary Figure 18.** Graph showing relative abundance correlations between RT2-specific ARGs and bacterial genera. Only “gene—gene” and “gene—genus” pairs with high (> 0.7) Spearman’s correlation coefficients are shown.

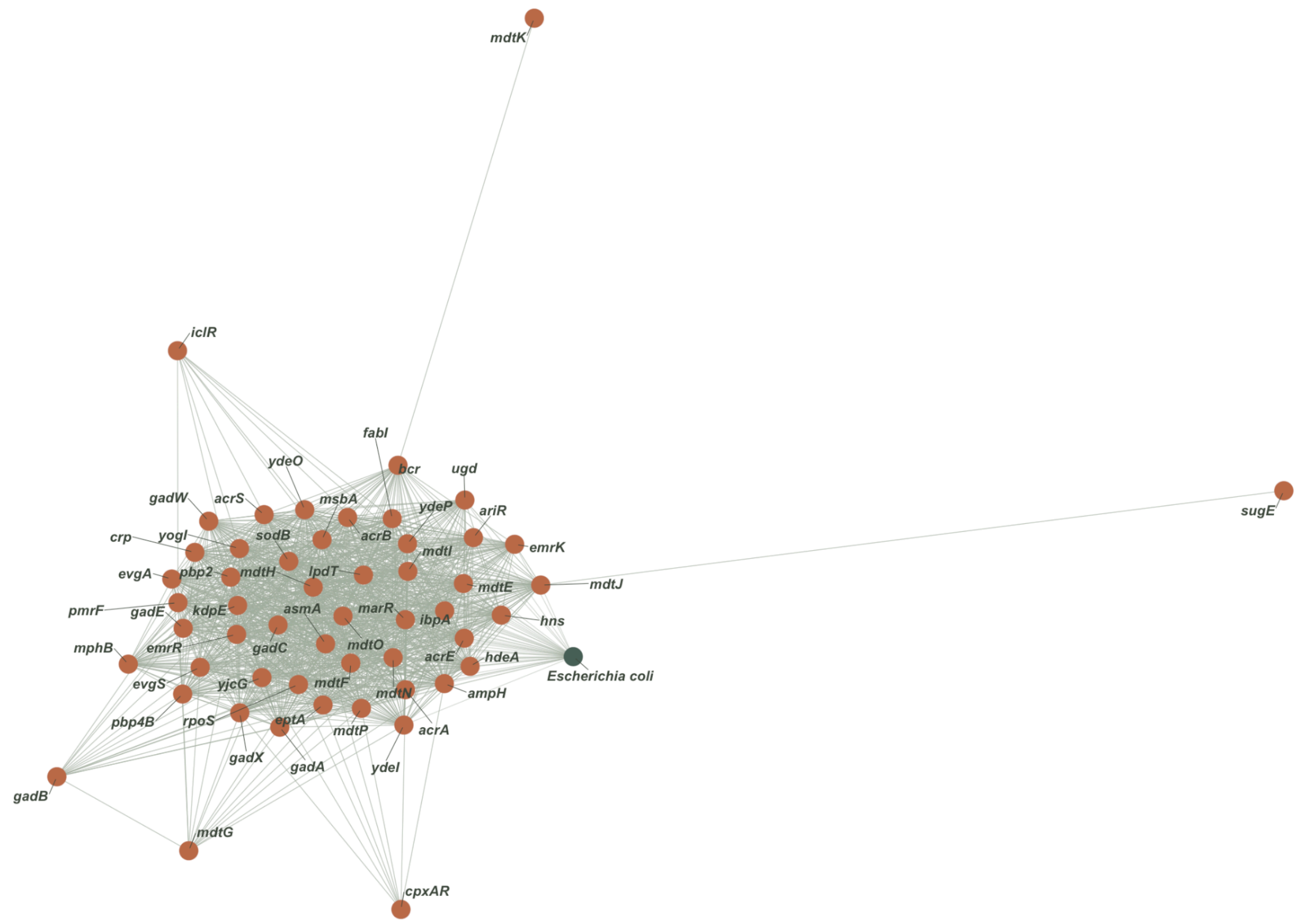


**Supplementary Figure 19.** Graph showing relative abundance correlations between RT2-specific ARGs and bacterial species. Only "gene—gene" and "gene—species" pairs with high (> 0.7) Spearman's correlation coefficients are shown.

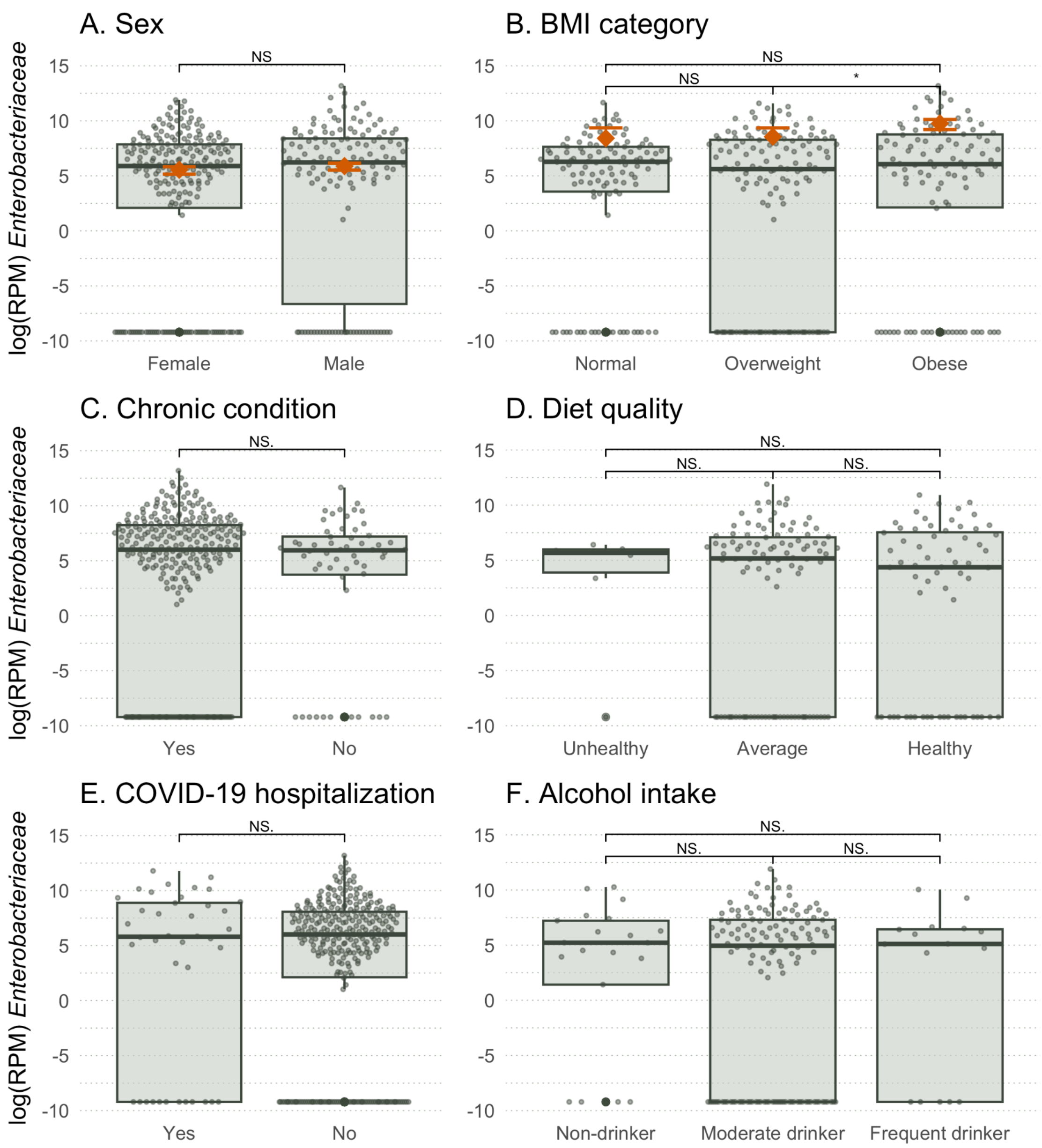


**Supplementary Figure 20.** *Enterobacteriaceae* relative abundances (log-transformed, pseudocounts added) in relation to **A**. sex (all samples considered, n = 350), **B**. BMI category (all samples considered, n = 350), **C**. the presence of a chronic condition (2016-2017 samples, n = 175), **D**. diet quality (2016-2017 samples, n = 175), **E**. COVID-19 hospitalization (2022 samples, n = 175), and **F**. the frequency of alcohol intake (2016-2017 samples, n = 175). For **A** and **B**, orange points and error bars represent estimated marginal means with 95% confidence interval from LMM (with year, sex, BMI, age group as fixed effects, and participant ID as random effect); significance levels are based on pairwise comparisons with Bonferroni correction. For **C-F**, significance levels are based on Mann-Whitney U test. * — p-value < 0.05; NS — p-value >0.050. Dots represent individual samples.
